# Force Generation by Enhanced Diffusion in Enzyme-Loaded Vesicles

**DOI:** 10.1021/acs.nanolett.5c00306

**Published:** 2025-03-26

**Authors:** Eike Eberhard, Ludwig Burger, César L. Pastrana, Hamid Seyed-Allaei, Giovanni Giunta, Ulrich Gerland

**Affiliations:** Physics of Complex Biosystems, Department of Bioscience, School of Natural Sciences, Technical University of Munich, 85748 Garching, Germany

**Keywords:** enzymes, vesicles, enhanced diffusion, chemotactic motion, microswimmer, self-propelled particles

## Abstract

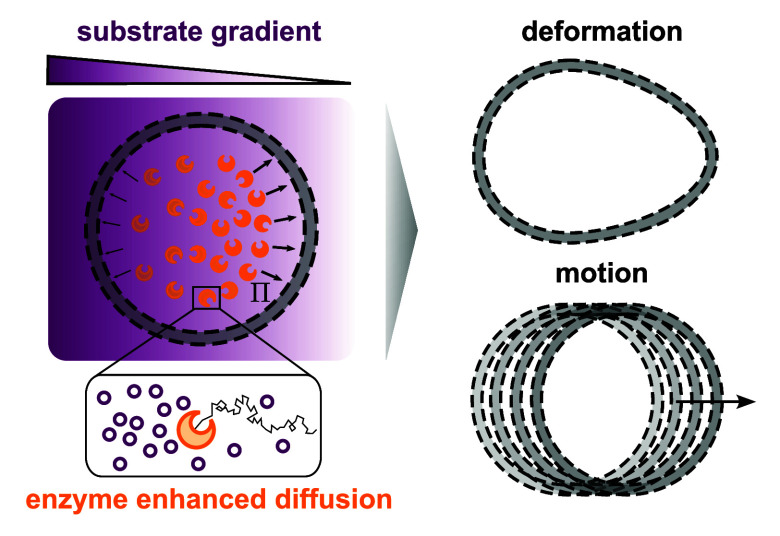

The diffusion coefficient of some metabolic enzymes increases with the concentration of their cognate substrate, a phenomenon known as enhanced diffusion. In the presence of substrate gradients, enhanced diffusion induces enzymatic drift, resulting in a nonhomogeneous enzyme distribution. Here, we study the effects of enhanced diffusion on enzyme-loaded vesicles placed in external substrate gradients using a combination of computer simulations and analytical modeling. We observe that the spatially inhomogeneous enzyme profiles generated by enhanced diffusion result in a pressure gradient across the vesicle, which leads to macroscopically observable effects, namely deformation and self-propulsion of the vesicle. Our analytical model allows us to characterize the dependence of the velocity of propulsion on experimentally tunable parameters. The effects predicted by our work provide an avenue for further validation of enhanced diffusion, and might be leveraged for the design of novel synthetic cargo transporters, such as targeted drug delivery systems.

In addition to catalyzing biochemical reactions, enzymes can exhibit properties akin to active matter. For instance, the effective diffusion coefficient of some enzymes increases in the presence of their cognate substrate, a phenomenon known as enhanced diffusion.^1−5^ While the effect of enhanced diffusion has been validated through several different experimental assays,^6−9^ the microscopic mechanism underlying this effect remains under debate:^10−14^ some attribute enhanced diffusion to nonpropulsive mechanisms,^7,15^ whereas others emphasize the role of dissipative propulsion,^9^ for instance, due to phoresis^1,2^ or pressure waves.^4^ Regardless of the microscopic mechanism, enhanced diffusion causes enzymes to drift from regions of high diffusivity (high substrate concentration) toward regions of low diffusivity (low substrate concentration), resulting in a nonhomogeneous enzyme distribution.^5,6^

Here, we propose a microdevice that uses enhanced diffusion of enzymes to deform and propel itself. Using coarse-grained simulations and analytical modeling, we demonstrate that enzymes encapsulated in a micron-sized vesicle can exert forces of a few piconewtons when they exhibit enhanced diffusion in a substrate gradient. These forces cause shape deformations, alter the fluctuation spectrum, and propel the vesicle with appreciable velocities.

Motile micro- and nanodevices hold significant potential for practical applications, particularly in the design of synthetic cargo transporters,^16,17^ such as drug delivery systems.^18−20^ Motion is usually achieved through slip velocities caused by phoretic effects or via the Marangoni effect.^16^ Phorectic effects may arise as a consequence of self-induced gradients, for example, from an inhomogeneous enzyme distribution on the surface of a moving bead^21−23^ or vesicle,^24^ or from an inhomogeneous permeability of a vesicle to product molecules produced inside.^18^ Marangoni effects are the result of a spatial variation of the surface tension of the membrane,^25^ which can be coupled to chemical reactions inside or outside the vesicle.^26−28^ In addition, propulsion can be achieved by encapsulating microswimmers in droplets or vesicles.^29^ Examples of microswimmers include flagellated bacteria,^30,31^ as well as self-propelled Janus particles.^32,33^ In both cases, the (hydrodynamic) interactions between the active swimmer and the boundaries of the container enhance the diffusive properties of the latter.^34^ The mechanism that causes the motion in our device is different: Enzymes encapsulated within the vesicle form a nonhomogeneous profile, creating a difference in osmotic pressure across the vesicle, which drives its deformation and motion. Unlike existing systems, this microdevice does not need to be inherently asymmetric: The asymmetry emerges dynamically, via enhanced diffusion of enzymes in an external substrate gradient.

Our results suggest that the morphological changes and the motion of enzyme-loaded vesicles should be detectable by light microscopy, providing an avenue for further validation of enhanced diffusion. Moreover, our analytical model rationalizes the dependence of the device’s behavior on enzyme properties and experimentally tunable parameters. This model could guide the design of synthetic biocompatible cargo systems with controllable active properties.

aining enzymes with cognate substrate *s*. The vesicle is exposed to an external substrate gradient, with substrate concentration  on the left end of the system, and substrate concentration *s*_*r*_ = 0 mM on its right end. Therefore, the substrate gradient is linear with a constant slope of /*L*, where *L* is the system size. The vesicle is assumed to be perfectly permeable to substrate and impermeable to enzymes (Figure 1). This assumption is justified by the (typically) much larger size of enzymes compared to that of their substrate. We neglect substrate depletion, such that the internal concentration of *s* is set by the external substrate gradient. Rapid permeation of substrate can be achieved by incorporating natural or artificial pores on the membrane^35,36^ (SI Sec. I), and imperfect permeability reduces the substrate concentration inside the vesicle by a constant permeability-dependent factor (SI Sec. I). We further assume that the enzymes undergo enhanced diffusive motion (Figure 1, inset), where the diffusivity of the enzymes is a function of the substrate concentration *s*(**r**) at position **r**,^13^
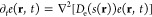
1

**Figure 1:**
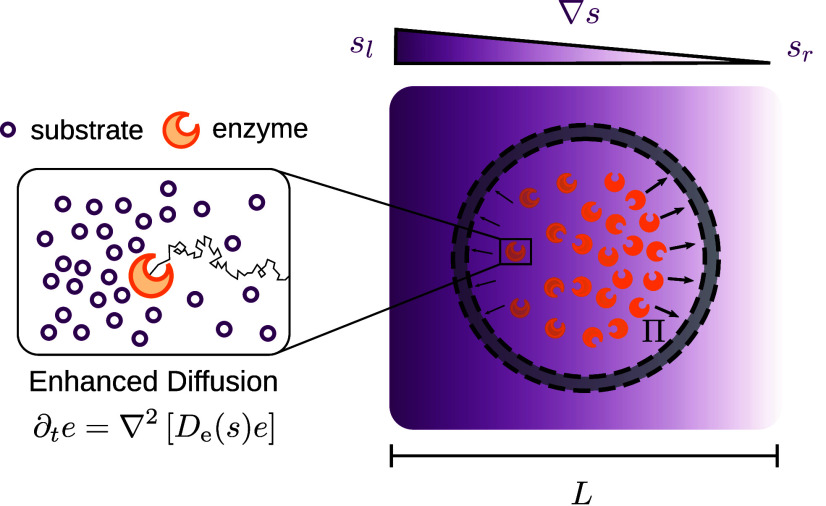
**Mechanism generating anisotropic pressure profile.** A vesicle is loaded with enzymes that catalyze the conversion of substrate *s*. While the substrate is assumed to freely permeate through the membrane, enzymes cannot pass the membrane. The vesicle is placed in an externally imposed substrate gradient (depicted in the figure by a purple shading), with nonzero substrate concentration  applied to the left end of the system, and vanishing substrate concentration on the system’s right end, *s*_*r*_ = 0 mM. *Inset:* The time-evolution of the enzyme is governed by an enhanced diffusion equation, i.e., the enzyme diffusion *D*_e_ is a function of the substrate concentration *s*. Enhanced diffusion causes the enzyme to move downstream substrate gradients (toward low substrate concentration). This results in a nonhomogeneous enzyme distribution within the vesicle and leads to an anisotropic pressure profile Π(**r**, *t*).

The effective diffusion coefficient *D*_e_(*s*) is defined as^4,6,37^
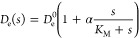
2

The factor α parametrizes the relative increase in diffusivity α=(*D*_e_^*max*^-*D*_e_^0^)/*D*_e_^0^ due to the substrate, with typical values in the range of 15–80% and an average of approximately 30%.^12^

In the presence of the substrate gradient, enhanced diffusion (eq 1) results in antichemotactic motion of the enzymes, causing the accumulation of enzymes in regions of low substrate concentration.^5,6^ Note that other studies report motion toward regions of high substrate concentration (chemotaxis),^1,37,38^ attributed to cross-diffusion contributions due to (nonspecific) interactions of enzymes with substrate and product molecules.^39,40^ However, cross-diffusion effects require very high substrate/product concentrations, and are neglected in our model. The spatially inhomogeneous enzyme profile created by antichemotaxis (eq 1) creates an anisotropic osmotic pressure on the membrane, Π(**r**, *t*) = *k*_B_*Te*(**r**, *t*), with *T* the temperature of the solvent.^33,41^ The pressure profile exerts a net force onto the vesicle,
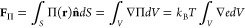
3where **n̂**(r) is the outward normal vector of the surface *S*, and *V* is the volume of the vesicle.

We estimate the magnitude of the force for a simplified scenario: (i) The substrate concentration is assumed to be a linear function of *x*, implying constant ∇*s*, (ii) the enzyme equilibration time scale is assumed to be small compared to the time scale associated with any possible deformation/displacement of the vesicle, and (iii) the vesicle is assumed to retain spherical shape. Using the steady state solution of eq 1, we obtain an equation for the force exerted by the enzymes (eqs S74 and S65 in SI). Enzymes with the properties of urease (α = 0.24, *K*_M_ = 3 mM, and *D*_e_^0^= 40 μm^2^ s^–1^) encapsulated in a vesicle of size R = 10 μm at a concentration of *e*_T_ = 100 nM and placed in a substrate gradient, ∇*s* = 0.05 mM /μm, exert a force of about **F**_Π_^(0)^≈1 pN. This force is in a biologically relevant range.^42^

We first characterize the deformation of the vesicle due to the pressure exerted by the enzymes. To this end, we simulate the dynamics of the system depicted in Figure 1 using a membrane model that is known to correctly capture the equilibrium fluctuation spectrum of lipid membranes and their deformation induced by active particles.^32,43−45^ The model accounts for bending elasticity, membrane fluidity, area conservation, and a volume constraint (SI Sec. II A and II B). To model the pressure profile exerted by the enzymes, Π(**r**, *t*), we introduce a repulsive short-range interaction between the vesicle surface and the enzymes. The motion of enzymes is described by an overdamped Langevin equation with multiplicative noise, which is equivalent to the enhanced diffusion equation eq 1 (SI Sec. II D).

Our simulations indicate that enzymes encapsulated in vesicles cause substantial deformations as a consequence of enhanced diffusion (Figure 2). The magnitude of the deformation depends on the osmolarity of the solution that surrounds the vesicle, i.e., the concentration of osmotically active solutes outside of the vesicle that exert osmotic pressure in addition to the enzymes. To account for the osmolarity of the solution, we incorporate a volume constraint into the vesicle model, which allows us to study the system under hyperosmotic (high concentration of osmotically active solutes) and hypoosmotic (low concentration of osmotically active solutes) conditions.^46^

**Figure 2:**
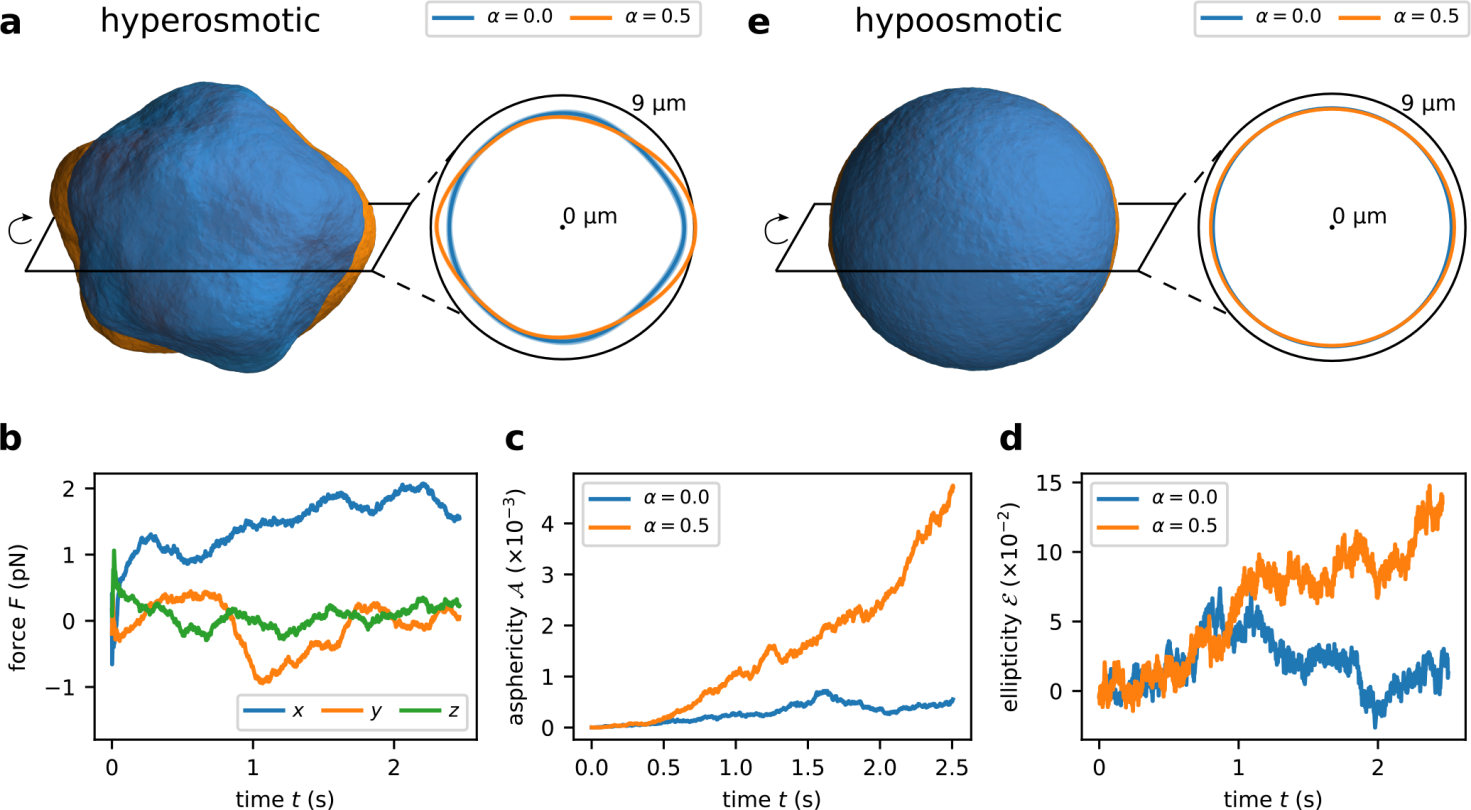
**Deformations of enzyme-loaded vesicles.** a)-d) Vesicles under hyperosmotic conditions (*k*_*V*_ = 129 N m^–2^, V̂ = 0.9) filled with enzymes that do (α = 0.5, orange) or do not exhibit enhanced diffusion (α = 0.0, blue) in the presence of an external substrate gradient. a) Snapshots of the vesicle shape (*left*) and averaged cross sections (*right*) show a prolate deformation of the vesicle due to enhanced diffusion. The averaged cross sections are obtained by averaging over multiple time frames and rotations along the gradient axis: The continuous lines represent the local average radius, while the standard deviation is shown as shaded area. b) Components of the force vector acting on vesicles under hyperosmotic conditions as a result of enzyme-vesicle interaction. The force component parallel to the direction of the gradient is nonzero. c)-d) The shape parameters aspheriticy  and ellipticity  in vesicles under hyperosmotic conditions are nonzero, indicating a prolate deformation of the vesicle. e) Shape of vesicles under hypoosmotic conditions (no volume constraint) filled with enzymes that do (α = 0.5, orange) or do not exhibit enhanced diffusion (α = 0.0, blue) in the presence of an external substrate gradient. Snapshots (*left*), averaged cross sections (*right*) and shape parameters (Figure S3) indicate a subtle prolate deformation due to enhanced diffusion. The parameters used in the simulations are summarized in SI Table 2.

Under hyperosmotic conditions, the vesicle shows clear deformations (including protrusions) even in the absence of enhanced diffusion (Figure 2a, left). Since only a two-dimensional projection of the three-dimensional shape can be observed in a typical light microscopy setup, we determine the shape of this projection by averaging over the different cross sections obtained through rotation around the gradient axis. The cross-section reveals a preferential stretching of the vesicle along the direction of the substrate gradient for α = 0.5 (Figure 2a, right). Other shape parameters of the vesicle (e.g., asphericity  or ellipticity ) also indicate a pronounced prolate deformation (Figure 2c,d, SI Sec. II F). Both  and  do not saturate within the simulated time, suggesting even larger deformations at longer times. The force generated along the *x*-axis in the presence of enhanced diffusion (α = 0.5) builds up, reaching values ≈2pN after 2s (Figure 2b), comparable to the estimate obtained above.

Under hypoosmotic conditions (Figure 2e), vesicles exhibit only slight deformations. Although the deformation is hard to detect by visual inspection, closer analysis of the cross-section reveals a subtle prolateness along the gradient axis. This is consistent with an ellipticity of –3% (Figure S3). The vesicle reaches a steady-state shape after approximately 1s, as indicated by the shape observables approaching a constant asymptotic value.

Since the analysis of membrane fluctuations is a well-established experimental technique to quantify the shape and mechanics of lipid membranes,^32,47−51^ we checked if the fluctuation spectrum of the vesicle’s radius reveals signatures of deformation (SI Sec. II G). We find that the fluctuation amplitudes  in the absence of enhanced diffusion (α = 0) agree perfectly with the theoretical prediction for membranes in equilibrium with a surface tension of σ = 0.97 μN m^–1^ (Figure 3a, SI Sec. III). In the presence of enhanced diffusion (α = 0.5), the spectrum exhibits significant deviations for the lowest modes  = 2 and  = 3. These modes correspond to elliptic and triangular deformations (Figure 3b), consistent with the shape observed in Figure 2e.

**Figure 3:**
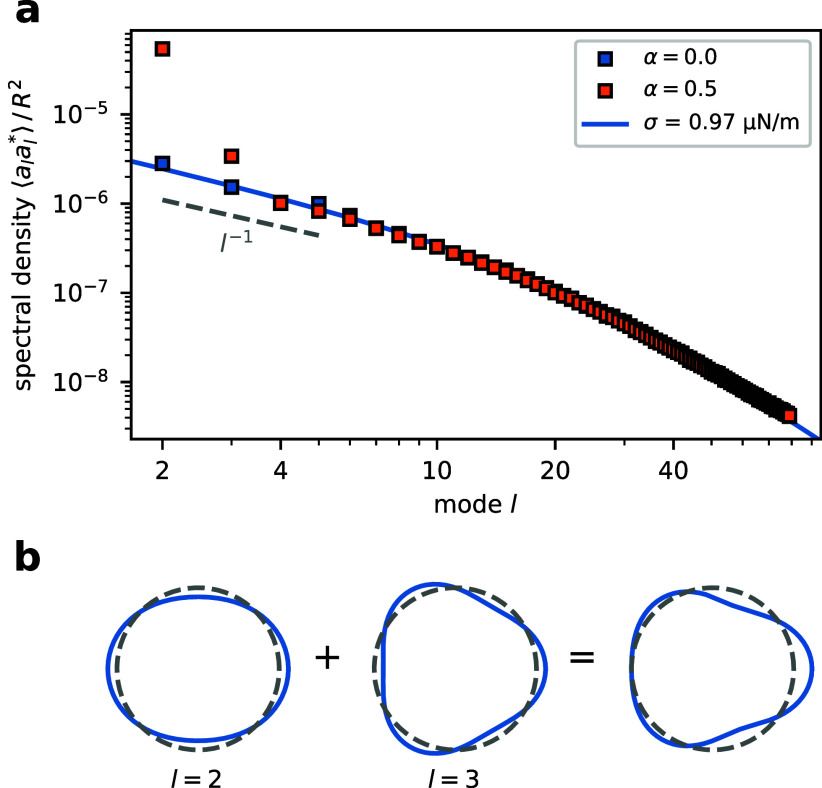
**Morphology of enzyme-loaded vesicles.** a) Fluctuation spectra of vesicles under hypoosmotic conditions (no volume constraint) filled with enzymes that do (α = 0.0, red) or do not display enhanced diffusion (α = 0.5, blue). The second and the third fluctuation modes are significantly more pronounced for α = 0.5 than for α = 0.0. The characteristic scaling in the tension-dominated regime  ∼ ^–1^ is highlighted as dashed line. The continuous blue line is the spectral density predicted by the model described in SI Sec. III. The parameters used in the simulations are summarized in SI Table 2. b) Schematic illustration of the deformations corresponding to the two fluctuation modes that are boosted by enhanced diffusion.

In addition to deformations, we observe that the vesicle propels itself through the externally imposed gradient. It is most straightforward to analyze this motion under hypoosmotic conditions, where deformations are negligible, such that contributions from shape deformation and translation to the displacement of the center of mass do not need to be disentangled. We therefore introduce a simplified model where the vesicle is treated as a perfect, nondeformable sphere subjected to Stokes’ friction (SI Sec. II C). Unlike the mesh-based model used before, the simplified simulation accurately captures vesicle friction (SI Sec. II H), predicting quantitatively correct propulsion velocities. The center of mass of the vesicle moves toward regions of lower substrate concentration. The vesicle translates at a velocity *v* = 0.61 μm s^–1^ (Figure 4), which is a factor ∼ 70 slower than the velocity expected based on the steady-state enzyme distribution *e*(***r***) (obtained by solving eq 1 in steady state) and its associated force on the vesicle **F**_Π_ (eq 3). Interestingly, the enzyme profile observed in the simulation is more homogeneous in space (Figure 4b) than the steady-state distribution predicted by eq 1, implying that the vesicle motion (and the associated drift of enzymes) alters the enzyme profile. This suggests that vesicle motion needs to be accounted for to predict the enzyme profile and the forces exerted on the membrane accurately.

**Figure 4:**
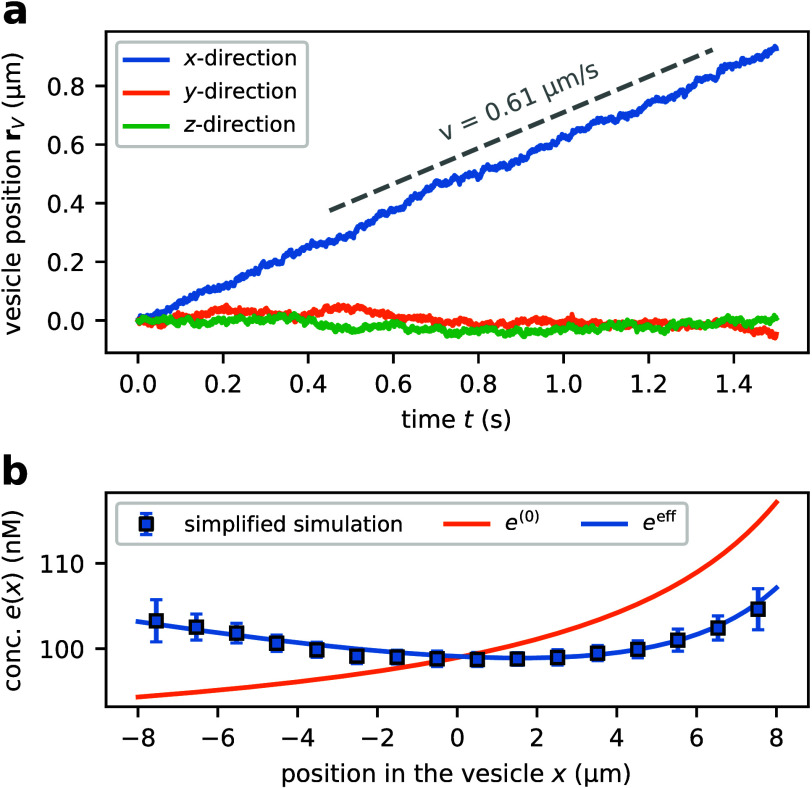
**Self-propulsion of enzyme-loaded vesicles.** a) Time-evolution of the vesicle position (center of mass) obtained via the simplified simulation. The vesicle moves downstream along the substrate gradient with velocity *v* determined via a linear fit. Vesicle trajectories obtained via the full mesh-based simulation are shown in Figure S4. b) Enzyme concentration profile along the gradient axis. The adiabatic profile *e*^(0)^ (steady-state solution of eq 1) is steeper than the effective trajectory-averaged profile *e*^eff^, which includes corrections to linear order in Péclet number. The simulated enzyme profile is obtained by averaging enzyme profiles observed along the trajectory for *t* ≥ 0.25 s (to avoid bias by the initial relaxation of the profile, SI Sec. V) and shows good agreement with the effective trajectory-averaged enzyme profile to linear order in Péclet number, *e*^eff^. The parameters used in the simulation are summarized in SI Table 3.

Hence, we describe enzyme diffusion in the comoving frame (vesicle at rest) by including an additional drift term in eq 1,
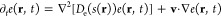
4where **v** is the velocity of the vesicle. The corresponding steady-state enzyme profile is a function of the dimensionless Péclet number, Pe = *Rv*/*D*_e_^0^, the time scale of diffusion relative to the time scale of vesicle drift. Even at the highest velocities observed in the simulation, *v* ≈ 0.6 μm s^–1^, for a vesicle with radius R = 8 μm and diffusion coefficient *D*_e_^0^ = 39 μm^2^ s^–1^, the Péclet number is small, Pe ≈ 0.12. This allows us to expand the steady-state enzyme profile in the Péclet number (SI Sec. IV). To zeroth order, we recover the steady-state profile and velocity obtained by solving eq 1, *v*^(0)^ = *F*_Π_^(0)^/(6*πηR*) (SI Sec. IV B). We refer to the limit Pe → 0 as the adiabatic limit, since the motion of the vesicle is much slower than the enzyme diffusion in this limit. The velocity to linear order in Péclet number *v*^(1)^ can be expressed in terms of the adiabatic velocity *v*^(0)^,
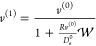
5with a non-negative weight  that depends on the dimensionless diffusion coefficient *D*_e_(*x*)/*D*_e_^0^ and on the vesicle radius *R*. The translation velocity is well-approximated by the adiabatic velocity,
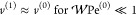
6provided the adiabatic Péclet number Pe^(0)^=*Rv*^(0)^/*D*_e_^0^, is small. In the opposite limit, the translation velocity approaches an asymptotic value independent of the adiabatic velocity,
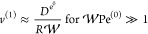
7

In both limits, the translation velocity of the vesicle depends on its position in the system. As the vesicle moves, the substrate concentration within the vesicle changes, affecting the effective diffusion coefficient of the enzymes, and thus the pressure and the ensuing translation velocity. Therefore, the velocity observed in the simulation corresponds to an effective velocity, averaged over the positions of the vesicle along its trajectory.

To directly compare the theoretical prediction to the simulations, we compute the effective velocity *v*^eff^ based on *v*^(1)^ by averaging *v*^(1)^(*x*_*V*_) over the vesicle trajectory *x*_*V*_(*t*) (SI Sec. IV C, neglecting the position dependence causes the analytical theory to underestimate the translation velocity). Analogously, the effective enzyme profile *e*^eff^(*x*) is defined as the average of the enzyme profiles along the trajectory of the vesicle, *e*^(1)^(*x*, *x*_*V*_), with *x* denoting a position within the vesicle (SI Sec. IV D). The analytically predicted translation velocities (Figure 4a, Figure 5) and enzyme profiles (Figure 4b) agree well with the simulation data.

**Figure 5:**
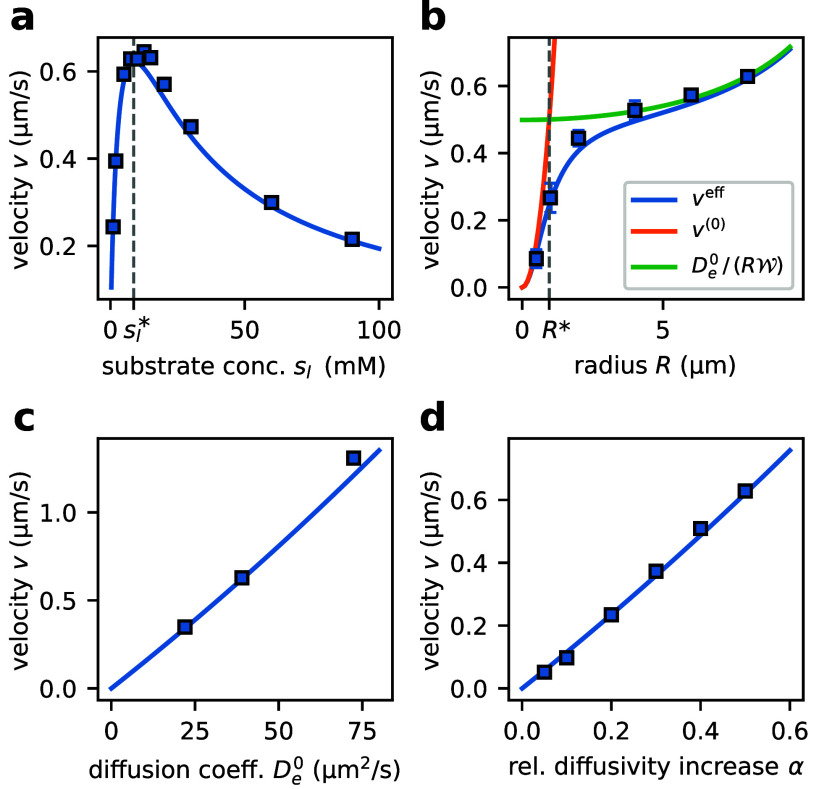
**Dependence of vesicle velocity on system parameters and enzymatic properties.** a) Vesicle velocity *v* depends nonmonotonously on the substrate concentration on the left boundary of the system , and exhibits a maximum at intermediate substrate concentration ( 8.7 mM, shown as dashed line). b) Velocity increases as a function of vesicle radius, with a quadratic radius-dependence for sufficiently small radii (smaller than R* ≈ 0.96 μm, shown as dashed line). c) Velocity increases linearly with the enzymatic basal diffusion coefficient *D*_e_^0^. d) Relation between the enhanced diffusion factor α and the velocity. All panels show the translation velocity in a medium with viscosity similar to that of water, η = 1 mPa s (parameters are summarized in SI Table 3). The dots represent the average of at least *n* = 10 simulations. The standard deviation of the mean is usually smaller than the marker size. The continuous curves show the effective trajectory-averaged velocity computed via the self-consistency approach to linear order in Péclet number. Figure S13 shows the parameter-dependence of velocity in a medium with high viscosity under otherwise identical conditions.

Figure 5a shows the velocity *v* as a function of the substrate concentration on the left boundary of the system, . The velocity depends nonmonotonically on , with a peak in velocity at around  ≈ 10 mM. This behavior is a consequence of the substrate-dependence of the diffusion coefficient *D*_e_(*x*). For small concentrations ( ≪ *K*_M_), the diffusion coefficient depends linearly on the substrate concentration. Thus, a steep substrate gradient enhances the difference in enzyme diffusivity between the left and right sides of the vesicle, leading to higher translation velocities. At saturating conditions ( ≫ *K*_M_), the diffusion coefficient is constant everywhere within the vesicle, which causes the pressure difference between left and right end of the vesicle (and consequently the translation velocity) to vanish. Solving for the substrate concentration  at which the velocity attains its maximum reveals that  depends linearly on *K*_M_,
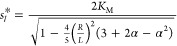
8(dashed line in Figure 5a and SI Sec. IV E). Note that  for vesicles that are far smaller than the system size, *R* ≪ *L*.

The radius-dependence of the velocity is shown in Figure 5b. Intuitively, the force *F* exerted by the enzymes is expected to be proportional to the number of enzymes in the vesicle, such that *F* ∝ *R*^3^ for fixed enzyme concentration. Together with Stokes’ law, the scaling of the force implies that the velocity should depend quadratically on the radius, *v* ∝ *R*^2^. This intuition is consistent with the quadratic radius-dependence of the adiabatic velocity *v*^(0)^ (SI Sec. IV F). As long as the vesicle radius is small enough for the adiabatic Péclet number Pe^(0)^ to be small, the translation velocity is dominated by the adiabatic velocity, and the velocity scales quadratically with *R* (orange curve in Figure 5b). As the radius increases, corrections beyond the adiabatic limit start to contribute significantly to the translation velocity. The characteristic radius R* beyond which corrections to the adiabatic limit are non-negligible is set by the condition ,
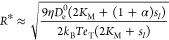
9(vertical dashed line in Figure 5b). In the limit of large radii, *R* ≫ R*, the translation velocity is given by the radius-dependent weight  (eq 7), implying , where *c*_*i*_ are radius-independent constants (green curve in Figure 5b, and SI Sec. IV F).

Enzyme properties also affect the translation velocity. We find that *v* depends linearly on the basal enzyme diffusion coefficient *D*_e_^0^. This is a consequence of Pe^(0)^ ≫ 1 over the entire range of diffusion coefficients (SI Sec. IV G): The translation velocity, eq 7, is proportional to *D*_e_^0^. We also investigate the effect of α on the velocity (Figure 5d), finding that *v* is proportional to α. This can be rationalized by noting that increasing α (and thus the effective diffusion coefficient) leads to a decrease of the diffusion time scale in a similar way as increasing *D*_e_^0^ does.

Numerous studies established^2−5^ and validated^6,8,9^ that certain enzymes exhibit enhanced diffusion in the presence of their substrate. Here, we showed that enhanced diffusion permits to design micron-sized devices capable of self-propulsion: Enzymes are encapsulated within a vesicle (Figure 1) and establish a spatially inhomogeneous enzyme profile due to antichemotactic drift caused by enhanced diffusion (Figure 4b). This enzyme profile generates a gradient of osmotic pressure across the vesicle, driving its deformation (Figures 2, 3) and propulsion (Figures 4, 5). While the prolate deformation is clearly visible under hyperosmotic conditions (Figure 2a-d), deformations are less pronounced in hypoosmotic conditions (Figure 2e). The vesicle propels itself with velocities on the order of 1 μm s^–1^ (Figure 4). We characterized the parameter-dependence of the velocity using an analytical model (Figure 5): The velocity depends linearly on the bare enzymatic diffusion coefficient, *D*_e_^0^, or the strength of enhanced diffusion, α (Figure 5c-d), and it increases nonlinearly with vesicle radius, *R*, or substrate gradient (Figure 5a-b). Substrate concentrations exceeding *K*_M_ can decrease the propulsion velocity due to enzyme saturation.

Recent advances in synthetic biology enable the construction of enzyme-loaded vesicles (SI Sec. VI): Vesicles of up to 100 μm in radius can be prepared,^52^ and, once loaded with enzymes, placed in a substrate gradient produced, e.g., by a microfluidic device.^1,6,53^ Microfluidic setups combined with bright-field/fluorescence microscopy can distinguish the propulsion mechanism described here from motion generated by other mechanisms. We focused on propulsion by the osmotic pressure gradient generated by enhanced diffusion, but we neglected hydrodynamic effects such as diffusiophoretic effects^23,28,54,55^ and Marangoni flows.^16,28,56^

Diffusiophoretic effects are generated by nonspecific interactions between colloidal particles and solute gradients. They can affect our system in two ways. First, substrate gradients outside of the vesicle can result in a slip velocity on the vesicle surface.^57^ This mechanism can be studied in control experiments with empty vesicles. Second, the motion of enzymes within the vesicle can be affected by phoretic effects arising from nonspecific interactions between enzymes and substrate/product molecules (cross-diffusion).^39,40^ There, the direction of motion of the enzymes is governed by the details of the nonspecific interactions, allowing for a switch from antichemotactic motion (due to enhanced diffusion) to chemotactic motion (due to cross-diffusion). One could perform control experiments with vesicles encapsulating enzymes with impaired enhanced diffusion (α ≈ 0), to isolate the contribution of enhanced diffusion to vesicle motion.

Marangoni flows are the result of surface tension gradients. Out-of-equilibrium chemical reactions can drive gradients in surface tension, for example by causing spatial inhomogeneities in the lipid composition,^26,27,58^ and similar effects might occur for enzyme-loaded vesicles. Control experiments without enzymes but with controlled substrate (or product) gradients could quantify the Marangoni contribution to the overall vesicle velocity produced by the surface activity of either of these molecules modulating the interfacial tension.^28^

Motile nano- and microdevices exhibiting (anti)chemotaxis are important for the design of synthetic cargo transporters,^16^ such as biocompatible drug delivery systems.^18−20^ For example, the ability of nanoswimmers to penetrate the blood-brain barrier is enhanced by chemotaxis in a glucose gradient.^18^ Similarly, antichemotaxis in an oxygen gradient might guide swimmers toward the oxygen-depleted center of a tumor.^59^ Further research is needed to determine the experimental feasibility and practical applicability of enhanced-diffusion based microswimmers, given potential challenges such as the loss of enzymatic activity or degradation of the vesicle.^60^ Our analysis may help to navigate the vast space of tunable system parameters, and aid the rational design of such a device.

## Data Availability

The simulations were implemented using the JAX-MD library.^61^ Simulations were run on Nvidia RTX 4090 and A100 GPUs. Implementation details can be found in Supporting Information Sec. II. The code and execution instructions are available in the GitHub repository.^62^

## Supporting Information Available

Details of computational methodologies, analytical procedures, and supplementary discussions (PDF)
