# Supplementary material for: Force Generation by Enhanced Diffusion in Enzyme-Loaded Vesicles
Source: Nano Lett. 2025 Mar 26;25(14):5754–61. doi: 10.1021/acs.nanolett.5c00306 (PMC11987064; doi:10.1021/acs.nanolett.5c00306)
Supplement: Supplementary file 1 — nl5c00306_si_001.pdf [file nl5c00306_si_001.pdf]

Supporting Information
Force Generation by Enhanced Diffusion in Enzyme-Loaded Vesicles

Eike S. Eberhard,[†] Ludwig Burger,[†] Cesar Lopez Pastrana,[†]
Hamid Seyed-Allaei, Giovanni Giunta,[*] and Ulrich Gerland[*]
Physics of Complex Biosystems, Technical University of Munich, 85748 Garching, Germany

CONTENTS

	Page
I. Substrate Permeability through the Vesicle	2
II. Simulation	5
A. Mesh-based Vesicle Model	5
B. Mesh generation for the Mesh-based Vesicle Model	6
C. Simplified Vesicle Model	7
D. Dynamics of the System	7
E. Initialization of Enzyme Distribution	7
F. Analysis of Shape Parameters in the Mesh-based Model	8
G. Shape Fluctuation Analysis in the Mesh-based Vesicle Model	8
H. Translational velocity in the Mesh-based Vesicle Model	9
I. Analysis of Enzyme Distribution	10
J. Parameters	11
III. Fluctuation Spectrum of Enzyme-filled Vesicles	12
IV. Translational Velocity of Enzyme-filled Vesicles	14
A. Steady-State Enyzme Profile	14
B. Force and Velocity	15
C. Effective Trajectory-Averaged Velocity	17
D. Effective Trajectory-Averaged Enzyme Profile	18
E. Substrate-Dependence of the Velocity	19
F. Radius-Dependence of the Velocity	23
G. Dependence of the Velocity on the Diffusion Coefficient	24
V. Dynamics and Fluctuations of Enzyme Distribution	25
A. Relaxation of the Initial Enzyme Distribution	25
B. Fluctuations around the Steady-State Enzyme Distribution	27
VI. Ideas for Experimental Designs	30
A. Substrate Gradient	30
B. Vesicles Encapsulating Enzymes	30
C. Measuring Deformation and Motion	30
List of Figures	31
References	31

[†] These authors contributed equally to this work.
[*] These authors jointly supervised this work.
Corresponding authors: giovanni.giunta@tum.de, gerland@tum.de

I. SUBSTRATE PERMEABILITY THROUGH THE VESICLE

In our model, we assume that the vesicle is infinitely permeable to substrate molecules. To address the question, how finite substrate permeability affects the deformation and motion of the vesicle, we investigate the relation between the internal substrate gradient and the membrane's permeability. In steady state, Fick's second law of diffusion implies that $D_s \Delta s = 0$. The solution to this Laplace equation can be obtained via an expansion in spherical harmonics. We consider the equilibrium substrate profile for a substrate gradient parallel to the x-axis. The boundary conditions

$$s_{\text{out}}(\mathbf{r}) = \nabla s_\infty \mathbf{e}_x \cdot \mathbf{r} = \nabla s_\infty \, x \qquad \text{for } r \gg R \tag{S1}$$
$$J = \gamma_p \left[s_{\text{in}}(\mathbf{r}) - s_{\text{out}}(\mathbf{r}) \right] \qquad \text{for } r = R \tag{S2}$$

divide the system into two domains, the vesicle's interior and exterior. Here, $\nabla s_\infty \in \mathbb{R}$ is the external substrate gradient far away from the vesicle, and γ_p is the permeability. The flux J across the membrane is given according to Fick's first law,

$$J = -D_s \nabla s_{\text{in}} \cdot \mathbf{e}_r \Big|_{||\mathbf{r}||=R} = -D_s \nabla s_{\text{out}} \cdot \mathbf{e}_r \Big|_{||\mathbf{r}||=R} . \tag{S3}$$

We express $s_{\text{in}}(\mathbf{r})$ and $s_{\text{out}}(\mathbf{r})$ in spherical harmonics using the general ansatz for square-integrable solutions with azimuthal symmetry of the Laplace equation,

$$s(r,\theta) = \sum_{l=0}^{\infty} (A_l r^l + B_l r^{-l-1}) P_l(\cos\theta) \tag{S4}$$

where P_l is the l-th Legendre polynomial. The coordinate system is chosen such that $x = r\cos\theta$. The terms with a negative power of r inside the vesicle need to vanish since the substrate concentration is finite. Additionally, we note that the asymptotic behavior at large distances from the vesicle implies that on the outside all powers ≥ 2 of r and any constant terms must also vanish. Hence,

$$s_{\text{in}}(r,\theta) = \sum_{l=0}^{\infty} I_l r^l P_l(\cos\theta) \tag{S5}$$

$$s_{\text{out}}(r,\theta) = \nabla s_\infty r P_1(\cos\theta) + \sum_{l=0}^{\infty} O_l r^{-l-1} P_l(\cos\theta) . \tag{S6}$$

We plug our ansatz into the continuity equation (Eq. (S3)), using the identity $\nabla f \cdot \hat{e}_r \equiv \partial_r f$, and obtain

$$\sum_{l=1}^{\infty} I_l l R^{l-1} P_l = \nabla s_\infty P_1 - \sum_{l=0}^{\infty} O_l (l+1) R^{-l-2} P_l , \tag{S7}$$

which is equivalent to the conditions

$$0 = R^{-2} O_0 \tag{S8}$$
$$0 = I_1 - \nabla s_\infty + 2R^{-3} O_1 \tag{S9}$$
$$0 = l R^{l-1} I_l + (l+1) R^{-l-2} O_l \qquad \forall l \geq 2 . \tag{S10}$$

Next, we use boundary conditions (Eq. (S2)),

$$\nabla s_\infty R P_1 + \sum_{l=0}^{\infty} O_l R^{-l-1} P_l - \sum_{l=0}^{\infty} I_l R^l P_l = \frac{D_s}{\gamma_p} \sum_{l=1}^{\infty} I_l l R^{l-1} P_l, \tag{S11}$$

to derive the additional relations

$$0 = R^{-1} O_0 - I_0 , \tag{S12}$$

$$0 = \nabla s_\infty R + R^{-2} O_1 - \left(R + \frac{D_s}{\gamma_p} \right) I_1 , \tag{S13}$$

$$0 = R^{-l-1} O_l - \left(R^l + \frac{D_s}{\gamma_p} l R^{l-1} \right) I_l \qquad \forall l \geq 2 . \tag{S14}$$

We notice that both $I_0 = 0$ and $O_0 = 0$. To see that I_l and O_l vanish for all $l \geq 2$, we use Eq. (S14) and plug it into Eq. (S10),

$$O_l = \left(R^{2l+1} + \frac{D_s}{\gamma_p} l R^{2l} \right) I_l \qquad \forall l \geq 2 \tag{S15}$$

$$0 = \underbrace{\left(l R^{l-1} + (l+1) R^{-l-2} \left(R^{2l+1} + \frac{D_s}{\gamma_p} l R^{2l} \right) \right)}_{\geq 0} I_l \,. \tag{S16}$$

We utilize the two remaining conditions,

$$O_1 = \frac{R^3}{2} \left(\nabla s_\infty - I_1 \right) \tag{S17}$$

$$0 = \nabla s_\infty R + \frac{R}{2} \left(\nabla s_\infty - I_1 \right) - \left(R + \frac{D_s}{\gamma_p} \right) I_1 \tag{S18}$$

to determine

$$I_1 = \frac{\nabla s_\infty}{1 + \frac{2}{3R} \frac{D_s}{\gamma_p}} \,, \tag{S19}$$

which implies the following solution for the substrate gradient,

$$s_{\text{in}}(r, \theta) = \frac{\nabla s_\infty}{1 + \frac{2}{3R} \frac{D_s}{\gamma_p}} r P_1(\cos\theta) \tag{S20}$$

$$s_{\text{out}}(r, \theta) = \nabla s_\infty \, r P_1(r \cos\theta) + \nabla s_\infty \frac{R^3}{2} \frac{\frac{2D_s}{3R\gamma_p}}{1 + \frac{2D_s}{3R\gamma_p}} \frac{P_1(\cos\theta)}{r^2} \tag{S21}$$

For the internal substrate gradient to match the outside gradient, $\nabla s_{\text{in}} \approx \nabla s_{\text{out}} = \nabla s_\infty$, the permeability γ_p must be sufficiently large,

$$\gamma_p \gg \frac{2D_s}{3R} \,. \tag{S22}$$

For urea ($D_s = 1.38 \times 10^{-9} \text{m}^2/\text{s}$ [1, 2]) in a vesicle of radius $R = 8\,\mu\text{m}$, the right hand side evaluates to $\frac{2D_s}{3R} \approx 10^{-4} \text{m/s}$. The solubility of urea in lipids is low, implying low permeability through lipid bilayers [3]. Typical values for the permeability of urea for these artificial membranes are in the order of $\gamma_p = 10^{-8}$ m/s (Table 1).

Pores can be added to the membrane to increase the permeability with regard to both water and substrate [4]. We approximate the permeability of a membrane with pores,

$$\gamma_p^s = \frac{A_{\text{pores}}^{(\text{in})}}{A_{\text{mem}}} \frac{D_s}{\delta_{\text{pore}}} + \frac{A_{\text{mem}} - A_{\text{pores}}^{(\text{out})}}{A_{\text{mem}}} \gamma_{p_0}^s \tag{S23}$$

$$= N_{\text{pores}} \frac{(r_{\text{pore}} - r_s)^2}{4R^2} \frac{D_s}{\delta_{\text{pore}}} + \left(1 - N_{\text{pores}} \frac{(R_{\text{pore}} + r_s)^2}{4R^2} \right) \gamma_{p_0}^s \,, \tag{S24}$$

where $A_{\text{pores}}^{(\text{in})}$ and $A_{\text{pores}}^{(\text{outer})}$ are the effective area of the inner channel and the area of the whole pore on the membrane, respectively. These can be approximately calculated using the inner and out pore radii r_{pore} and R_{pore}, and the effective radius of the substrate r_s. The total length of the channel through which the substrate can freely diffuse is given by δ_{pore}. Fig. S1a shows an illustration of the variables appearing in Eq. (S24).

In the following, we consider α-Hemolysin as an example for pores in the membrane. It has an inner pore radius of $r_{\text{pore}} = 13\,\text{Å}$ and a total channel length of $\delta_{\text{pore}} = 100\,\text{Å}$ with an outer radius of approximately $R_{\text{pore}} = 50\,\text{Å}$ [5]. For urea, we assume an effective radius of $r_s^{(\text{urea})} = 2.2\,\text{Å}$ [6]. Using these parameters, we estimate the number of pores needed to satisfy the permeability criterion (Eq. (S22), Fig. S1b). For α-Hemolysin, the permeability criterion is met if the number of pores is around $N_{\text{pores}} \approx 2 \cdot 10^5$. For this pore number, approximately 2% of the membrane area is covered by α-Hemolysin. We suppose that this coverage by pores is experimentally feasible, as there is evidence

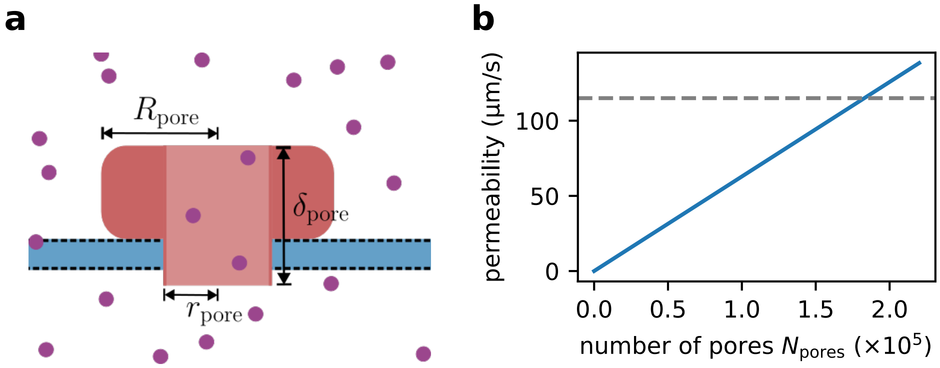

Figure S1. **Pore-dependence of membrane permeability** a) Illustration of a membrane pore. b) The membrane permeability increases linearly with the number of pores according to Eq. (S24), and exceeds the characteristic permeability (Eq. (S22), dashed line) for sufficiently high number of pores. Here, we show the permeability of urea through a POPC vesicle with a radius of $R = 8\,\mu\text{m}$ with N_p α-Hemolysin pores (Table 1).

**Lipid**	Membrane Thickness δ[Å]	$\gamma_{p0}^{\text{water}}$[μm/s]	$\gamma_{p0}^{\text{urea}}$[μm/s]
**POPC**	37.0	130	0.013
**DPPC**	28.4	2600 (at 50 °C)	0.3 (at 50 °C)
**DOPC**	28.8	158	0.0053

Table 1. **Properties of Artificial Membranes (Phosphatidylcholines)** [9–15]

indicating that membranes can sustain up to 33% coverage by α-Hemolysin without losing stability [7]. Moreover, vesicles with larger radii would further relax the membrane's permeability requirements (Eq. (S22)). Giant lipid vesicles with radii of up to $R \approx 15\,\mu\text{m}$ have been realized in similar experimental settings [8].

For vesicles that are not perfectly permeable to the substrate (e.g., due to an insufficient number of pores in the membrane), the substrate concentration within the vesicle is reduced by a constant permeability-dependent factor compared to the substrate concentration outside of the vesicle (Eq. S22). Consequently, the average substrate concentration within the vesicle is lower and the gradient of substrate within the vesicle is more shallow than in the case of a perfectly permeable vesicle (continuous line in Fig. S2a). Indeed, a vesicle with reduced permeability behaves identically to a perfectly permeable vesicle in a weaker external substrate gradient (dashed lines in Fig. S2a), which is equivalent to rescaling the concentration-axis in the plot of velocity vs. substrate concentration (Fig. S2b). The velocity shown in this plot is computed analytically as discussed in Sec. IV).

In addition, changes in the vesicle's permeability to the solvent impact the viscous force exerted by the surrounding medium. Stokes' law relates the drag force F acting on an impermeable sphere with a non-slip boundary condition to its translation velocity, $F = 6\pi\eta R v$, but the viscous drag force has experimentally been shown to be lower for permeable vesicles [16]. This implies that propulsion velocities observed in experiments are likely to be higher than the conservative estimate provided by our simulation.

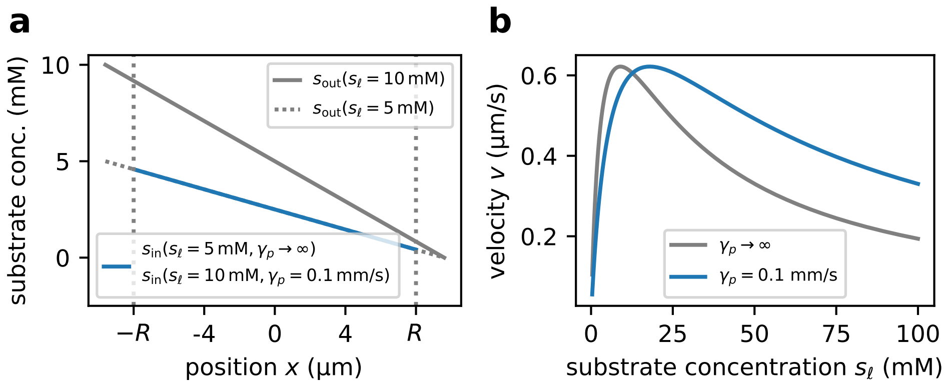

Figure S2. **Effect of reduced permeability to substrate.** a) Decreasing the vesicle's substrate permeability to substrate lowers the internal substrate concentration s_{in} in the vesicle (blue line between $-R$ and R) by a constant factor compared to the externally applied substrate profile $s_{\mathrm{out}}(s_\ell = 10\,\mathrm{mM})$. The resulting substrate profile for reduced permeability ($\gamma_p = 0.1\,\mathrm{mm\,s^{-1}}$) matches that of a perfectly permeable vesicle in a weaker external gradient (dotted line, $s_{\mathrm{out}}(s_\ell = 5\,\mathrm{mM})$. Thus, reducing permeability is equivalent to rescaling the substrate concentration applied at the system's left boundary, s_ℓ. b) The vesicle's translation velocity as a function of substrate concentration follows the same trend as in the perfectly permeable case, with the only difference being a rescaling of the externally applied substrate concentration s_ℓ. System parameters are summarized in Table 3

II. SIMULATION

A. Mesh-based Vesicle Model

The vesicle is simulated using a coarse-grained dynamically triangulated mesh consisting of N_V vertices [8] (SI Sec. II B). The Helfrich bending energy is described by the well-known equation $U_b = 2\kappa \iint H^2\,\mathrm{d}S$, where H is the mean curvature, κ is the bending modulus [17]. This energy is discretized on the mesh [18],

$$U_b = \frac{\kappa}{2}\sum_{i=1}^{N_V}\frac{1}{\sigma_i}\left[\sum_{j\in\mathrm{neigh.}(i)}\frac{\sigma_{ij}}{r_{ij}}\mathbf{r}_{ij}\right]^2, \tag{S25}$$

where the sum runs over all vertices N_V and $\mathbf{r}_{ij}$ vector from node j to node i. The term σ_{ij} corresponds to the length of the bond in the dual lattice $\sigma_{ij} = \frac{r_{ij}}{2}(\cot\theta_1 + \cot\theta_2)$, where θ_1 and θ_2 are the angles opposite to the bond i-j. The term σ_i is a normalization factor and accounts for the total area of the dual cell around vertex i and is given by $\sigma_i = \frac{1}{4}\sum_{j\in\mathrm{neigh}(i)}\sigma_{ij}r_{ij}$.

The area of the vesicle membrane is fixed by harmonic potential acting locally on the triangles of the mesh [8, 19]:

$$U_{\Delta\text{-area}} = \frac{k_s}{2}\sum_{i=1}^{N_T}\frac{(A_i - A_0)^2}{A_0}, \tag{S26}$$

where A_i are the instantaneous triangle areas, $A_0 = \frac{4\pi R^2}{N_T}$ and k_s is the area stretching stiffness. The sum runs over all triangles $N_T = 2(N_V - 2)$ [8, 19].

To account for osmotic effects [20], when specified, we use the harmonic potential

$$U_{\mathrm{V}} = \frac{k_V}{2}\frac{(V - V_t)^2}{V_0} \tag{S27}$$

to constrain the volume, where k_V is the volume stiffness and V_t is the target volume of the vesicle. We define $\hat{V} \equiv V_t/V_0$, where $V_0 = \frac{4}{3}\pi R_0^3$. The volume enclosed by the non-convex polyhedron is determined as described in [21, 22].

In the absence of volume constraints, the pressure exerted by the enzymes (both with and without enhanced diffusion) inflates the vesicle, stretching the surface: the vesicle is in a high-tension state. Conversely, introducing a volume

constraint mimics hyper-osmotic conditions (by setting a target volume $\hat{V} \equiv V/V_0 < 1$), which acts against the pressure exerted by the enzymes and the vesicle is at low-tension conditions.

The stability of the mesh is maintained by the bond potential

$$U_{\text{bond}}^{\text{rep}}(r) = \begin{cases} k_b^{\text{rep}} \frac{l_b}{r - l_{\min}} \left[1 - \exp\left(\lambda^{\text{rep}} \frac{r - l_{c1}}{l_b}\right)\right]^4 & \text{if } r > l_{c_0} \\ 0 & \text{else,} \end{cases} \tag{S28}$$

$$U_{\text{bond}}^{\text{att}}(r) = \begin{cases} k_b^{\text{att}} \frac{l_b}{l_{\max} - r} \left[1 - \exp\left(\lambda^{\text{att}} \frac{l_{c0} - r}{l_b}\right)\right]^4 & \text{if } r < l_{c_1} \\ 0 & \text{else.} \end{cases} \tag{S29}$$

Here, k_b^{rep} and k_b^{att} are the bond stiffness parameters. The values $l_{\min}$ and $l_{\max}$ set the minimum and maximum bond lengths allowed, and the constants λ^{rep} and λ^{att} control how rapidly the potentials increase between the cutoff and the limiting bond lengths. The parameters l_{c0} and l_{c1} are the cutoff lengths of the attractive and repulsive term of the bond potential, respectively, and thus, membrane vertices can move freely in the range $r \in [l_{c_1}, l_{c_0}]$.
To account for the fluidity of lipid bilayers, we allow the triangulation to change dynamically [19, 23]. In this procedure, the edges shared by adjacent triangles can be flipped. If a flip occurs, the bond along the shared edge is replaced by a bond connecting the previously unconnected vertices. A randomly selected fraction ψ of all bonds is tested at a frequency of ω. The parameters ω and ψ define the viscosity of the membrane [19], and we employed the values described in [8]. To preserve the stability of the mesh, we discard from bond flipping vertices of the shared edge with coordination number ≤ 5. Finally, a bond flip trial is accepted or rejected following the Metropolis criterion with probability $p_{\text{flip}} = \min\left\{\exp\left(-\frac{\Delta U}{k_B T}\right), 1\right\}$, where $\Delta U = U_{\text{flipped}} - U_{\text{not flipped}} \approx \Delta U_{\text{bond}} + \Delta U_{\Delta\text{-area}}$.
Enzymes are described as particles that do not interact with each other. The interaction between enzymes and the vertices of the membrane is harmonic and of the form

$$U_{\text{enz-mem}} = \frac{\epsilon}{2} \sum_{i=1}^{N_V} \sum_{j=1}^{N_{\text{enz}}} \left(1 - \frac{r_{ij}}{\sigma_{\text{enz}}}\right)^2 \Theta(\sigma_{\text{enz}} - r_{ij}) \tag{S30}$$

Here $r_{ij} = \|\mathbf{r}_{\text{mb},i} - \mathbf{r}_{\text{enz},j}\|$ is the distance between the coordinates of the enzymes and the membrane vertices and the sum runs over all the N_V vertices and all the N_{enz} pairs. The Heavyside function Θ restrains the potential to distances $r < \sigma_{\text{enz}}$.

B. Mesh generation for the Mesh-based Vesicle Model

While many strategies to initialize the membrane mesh make use of the spherical geometry to formulate a structured approach in building a triangulation, this ansatz would lead to an unphysical long-ranged order in the membrane. For this reason, we choose a stochastic growth process. First, all vertices are sampled from a uniform distribution on the unit sphere. The resulting positions are then iteratively relaxed using gradient descent with respect to different potential energy functions. We start by applying a radial restraint term $U_{\text{radial restraint}}^{(i)} \propto (r_i - R)^6$, with R the targeted equilibrium radius of the vesicle. This is combined with a repulsive soft sphere interaction potential acting between the membrane particles $U_{\text{mem-mem}}^{(\text{init})} \propto \left(1 - \frac{r}{\sigma_{\text{mem-mem}}^{(\text{init})}}\right)^4 \Theta(\sigma_{\text{mem-mem}}^{(\text{init})} - r)$, with $\sigma_{\text{mem-mem}}^{(\text{init})} \approx 2l_{\max}$. This process is run until a visually satisfying spacing between points is achieved. After this first crude relaxation step, we use the *Advancing Front Surface Reconstruction* routine based on Delaunay triangulation [24] implemented in the *Computational Geometry Algorithms Library* [25] to construct a triangulated mesh from the unstructured point cloud of vertices. This triangulation would be numerically unstable if it was combined with the simulation bond potential directly. For this reason, we further relax the triangulation with a harmonic bond potential $U_{\text{harmonic-bond}}^{(ij)} \propto (r_{ij} - l_b)^2$ and the area potential $U_{\Delta\text{-area}} = \frac{k_A}{2} \sum_{i=1}^{N_T} \frac{(A_i - A_0)^2}{A_0}$ used in the simulation (for the relaxation, the strength of the potential, k_A, is chosen smaller than later in the simulation). In the process of relaxation, we allow for a reconfiguration of the triangulation using the bond flipping procedure at a temperature of $T = 0$K. In the final relaxation steps, we successively add the bending potential and the volume potential and replace the harmonic bond potential with a cubic one $U_{\text{cubic-bond}}^{(ij)} \propto (r_{ij} - l_b)^3$, while increasing the area conservation coefficient k_A to the correct value. After a sufficient number of steps, this results in a triangulation with very homogeneous bond lengths and triangle areas at a temperature of $T = 0\,$K, such that we can safely replace the harmonic potential with the correct bond potential.

This completes the membrane potential in the simulation. The resulting membrane state is then equilibrated to a temperature of $T = 300\text{K}$. For the simulation, the cubic bond potential is replaced by the bond potential introduced in Eq. (S29).

C. Simplified Vesicle Model

In the simplified simulation, the vesicle is modeled as a non-deformable sphere centered. The vesicle is uniquely defined by its center $\mathbf{r}_{\text{mem}}$ (which evolves in time) and its (time-independent) radius, and there is no need to represent the vesicle membrane as a mesh of vertices. The vesicle membrane interacts with the enzymes via the potential,

$$U_{\text{enz-mem}} = \frac{k_{\text{ves}}}{2} \sum_{i=1}^{N_{\text{enz}}} (r_i - R)^2 \Theta(r_i - R) \,, \tag{S31}$$

where $r_i = \|\mathbf{r}_{\text{enz},i} - \mathbf{r}_{\text{mem}}\|$ and k_{ves} is the vesicle stiffness. This potential confines all the enzymes within a spherical volume of radius R.

D. Dynamics of the System

For both vesicle models (mesh-based and simplified), we model the motion of enzymes by an overdamped Langevin approach (Brownian Dynamics),

$$\frac{d\mathbf{r}_i}{dt} = -\frac{D_i}{k_{\text{B}}T} \nabla_i U + \sqrt{2D_i}\boldsymbol{\xi}(t) \,, \tag{S32}$$

where $\mathbf{r}_i$ are the coordinates of the particle i. The equation accounts for the random forces due to the collisions with the surrounding fluid (modeled as Gaussian white noise), as well as for a drift due to the potential U. Here, the relevant potential is the membrane-enzyme interaction potential $U_{\text{enz}-\text{mem}}$. The diffusion coefficient of the enzymes is a function of the local substrate concentration and, consequently, a function of space $D_{\text{e}} = D_{\text{e}}(s(\mathbf{r}_{\text{e}}))$ (Eq. 2). We consider a linear substrate profile of the form

$$s(x) = \begin{cases} s_\ell, & \text{if } x < -L/2 \\ s_\ell \left(\frac{1}{2} - \frac{x}{L}\right), & \text{if } -L/2 \le x \le L/2 \\ 0, & \text{if } x > L/2 \,, \end{cases} \tag{S33}$$

with the vesicle initially placed at the origin $x = 0$. The length of the system L is chosen to be 20% longer than the largest simulated vesicle.

In the mesh-based vesicle model, the vertices follow the same Langevin equation as the enzymes. The vertex diffusion coefficient is different from the enzyme diffusion coefficient ([8] and Table 2) and a potential U that includes all contributions to the membrane energy listed in the explanation of the mesh-based vesicle model. In the simplified vesicle model, there are no vertices to represent the membrane. Instead, the center of mass follows the Langevin equation Eq. (S32) with a diffusion coefficient set by the radius of the vesicle and the viscosity of the surrounding medium, $D_{\text{mem}} = k_{\text{B}}T/(6\pi\eta R)$.

In all models, the equations of motion are integrated using the first-order Euler-Maruyama method. The initial enzyme distribution is chosen to be homogeneous or to follow the adiabatic enzyme profile $e^{(0)}$ (steady state solution of Eq. 1) depending on the context (SI Sec. II E).

E. Initialization of Enzyme Distribution

In the mesh-based simulation, the enzymes are initialized following the adiabatic steady-state distribution (Sec. IV A). The enzymes are placed within a slightly smaller R than the vesicle to avoid numerical instabilities associated with the repulsion force between enzymes and the vertices of the mesh.

In the simplified simulation, the positions of the enzymes are initialized randomly, resulting in a homogeneous distribution within the volume of the vesicle. Control runs with adiabatic steady-state initialization revealed no dependence of the results on the initial distribution of the enzymes when allowing for sufficient relaxation times.

F. Analysis of Shape Parameters in the Mesh-based Model

To characterize the shape of the vesicle (and the change in shape due to enhanced diffusion), we resort to a set of order parameters defined in terms of the gyration tensor,

$$Q_{\alpha\beta} = \frac{1}{N}\sum_{i=1}^{N_V}(\mathbf{r}_\alpha^{(i)} - \mathbf{R}_\alpha^{(i)})(\mathbf{r}_\beta^{(i)} - \mathbf{R}_\beta^{(i)}), \tag{S34}$$

where α and β denote the components x, y, z of position $\mathbf{r}^i$ of the ith mesh vertex. The sum runs over all the N_V vertices. The term $\mathbf{R}$ is the center of mass of the vesicle. Via diagonalization, the tensor $\mathbf{Q}$ is uniquely defined by its eigenvectors $\hat{\mathbf{e}}_1$, $\hat{\mathbf{e}}_2$, $\hat{\mathbf{e}}_3$ and the associated eigenvalues $\lambda_1 \leq \lambda_2 \leq \lambda_3$.

The asphericity $\mathcal{A}$ quantifies the degree of non-spherical distribution of the mass. It is defined as [19, 26, 27]

$$\mathcal{A} \equiv \frac{(\lambda_1 - \lambda_2)(\lambda_2 - \lambda_3)(\lambda_3 - \lambda_1)}{2(\lambda_1 + \lambda_2 + \lambda_3)^2}. \tag{S35}$$

The asphericity is normalized such that $0 \leq \mathcal{A} \leq 1$, where $\mathcal{A} = 0$ zero indicates a perfect spherical symmetry (not necessarily spherical in shape), and $\mathcal{A} = 1$ a one-dimensional distribution of mass (e.g., a cylinder).

The prolateness $\mathcal{P}$ allows to discriminate spheroids with prolate or oblate morphology [26],

$$\mathcal{P} \equiv \frac{(2\lambda_1 - \lambda_2 - \lambda_3)(2\lambda_2 - \lambda_1 - \lambda_3)(2\lambda_3 - \lambda_1 - \lambda_2)}{2(\lambda_1^2 + \lambda_2^2 + \lambda_3^2 - \lambda_1\lambda_2 - \lambda_1\lambda_3 - \lambda_2\lambda_3)^{3/2}} \tag{S36}$$

The prolateness is defined in the range $-1 \leq \mathcal{P} \leq 1$, where $\mathcal{P} < 0$ for oblate shapes and $\mathcal{P} > 0$ for prolate spheroids. We define the prolateness axis $\mathcal{P}_x = |\hat{\mathbf{e}}_1 \cdot \hat{\mathbf{e}}_x|$, where $\hat{\mathbf{e}}_1$ is the eigenvector associated to the eigenvalue λ_1 and $\hat{\mathbf{e}}_x$ is the orientation vector of the substrate gradient.

The observables described above rely on access to the 3D configuration of the vesicle. However, most experimental observations of vesicles use light microscopy imaging and can only capture the 2D cross-sections along the equatorial plane. We characterize the shape of the cross-section via its ellipticity,

$$\varepsilon = \frac{a}{b} - 1. \tag{S37}$$

Here, a denotes the length of the ellipse along the direction of the substrate gradient, while b denotes the length of the axis perpendicular to the direction of the gradient. We pick the direction along which b is measured such that b is maximal. A comparison of the shape parameters for vesicles under hyper- and hypoosmotic conditions is shown in Fig. S3.

G. Shape Fluctuation Analysis in the Mesh-based Vesicle Model

We characterize the morphology of the vesicle via shape parameters (SI Sec. II F) as well as via its fluctuation spectrum. To determine the latter, we conduct a Fourier analysis of the radial fluctuations of an equatorial cross-section of the vesicle [8, 28]. Vertices within a distance on the order of the bond length of the mesh l_b to the equatorial plane are orthogonally projected on that plane. The resulting 2D coordinates are transformed to polar coordinates $(x, y) \to (r, \theta)$, and linear interpolation is used to obtain N radial positions r at evenly spaced intervals for θ. The resulting radial positions are decomposed in Fourier modes,

$$a_\ell = \frac{1}{N}\sum_{m=0}^{N-1} r(\theta_m)\exp\left(\frac{-2\pi i\ell m}{N}\right), \tag{S38}$$

where $\theta_m = 2\pi m/N$ and ℓ is the mode number. We obtain the mean fluctuation spectrum by averaging spectra over N_ρ rotations and over a time window given by N_s uncorrelated frames,

$$\langle a_\ell \rangle = \frac{1}{N_\rho N_s}\sum_{s=1}^{N_s}\sum_{\rho=1}^{N_\rho} a_{\ell\rho s}\,. \tag{S39}$$

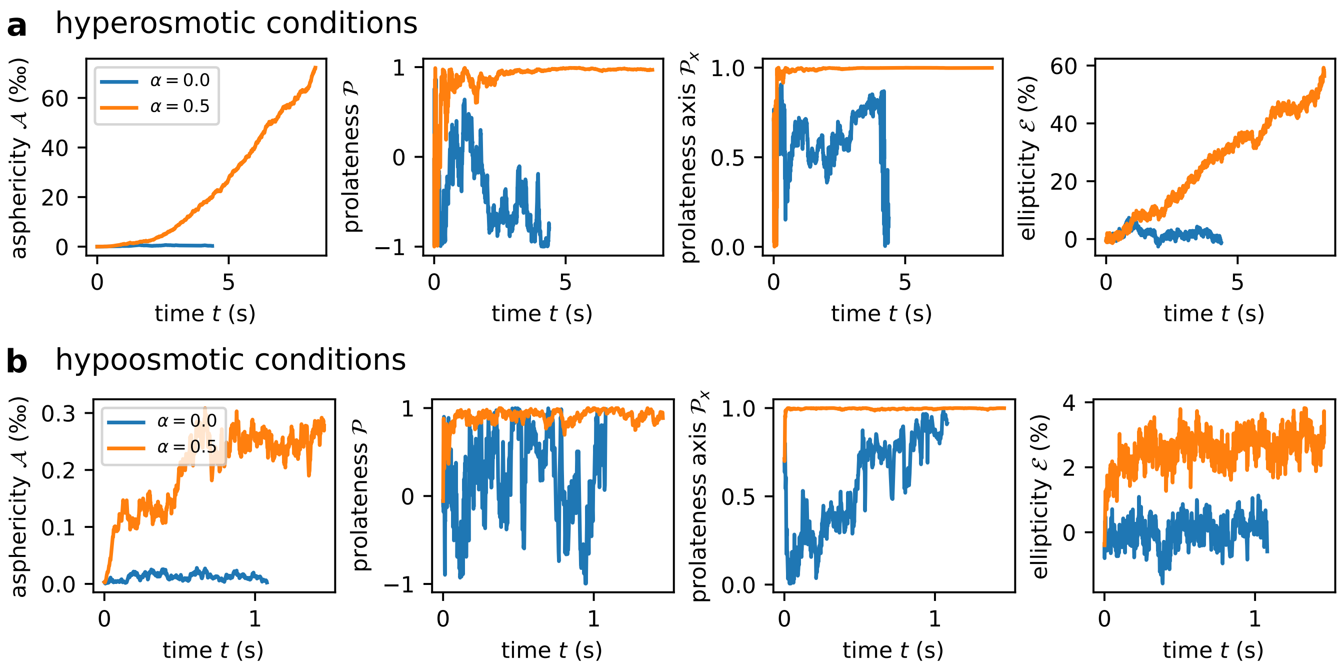

Figure S3. **Shape parameters of vesicles in hyper- and hypoosmotic conditions**. Asphericity $\mathcal{A}$, prolateness $\mathcal{P}$, prolateness axis $\mathcal{P}_x$ and ellipticity $\mathcal{E}$ for vesicles under hyperosmotic (a) and hypoosmotic conditions (b). In the case of hyperosmotic conditions, low surface tension is achieved by reducing the desired volume below the native sphere volume, $\hat{V} = V/V_0 = 0.9$ with volume stiffness $k_V = 129\,\mathrm{N\,m^{-2}}$. Under hypoosmotic conditions, no volume constraint is imposed, $k_V = 0\,\mathrm{N\,m^{-2}}$. While asphericity and ellipticity saturate in the case of hypoosmotic conditions, they continue to increase throughout the entire simulated time frame under hyperosmotic conditions. The parameters used in the simulations are summarized in Table 2.

The rotated configurations are computed using a linear transformation of the coordinates of the membrane vertices $\mathbf{r}_{\mathrm{mem}}$, following $\mathbf{r}'_{\mathrm{mem}} = \mathcal{R}_x \mathbf{r}_{\mathrm{mem}}$, where $\mathcal{R}_x = \mathcal{R}_x(\alpha)$ is the rotation matrix along the substrate gradient axis by an angle $\alpha \in [0, \pi)$. Note that a similar approach was used for the cross-section representations shown in Fig. 2a/e.

The illustrations in Fig. 3b are obtained via the inverse Fourier transform considering the following identity for a real-valued signal $r_\ell = 2\sum_{m=0}^{\lceil (N-1)/2 \rceil} \mathrm{Re}(a_m)\cos(2\pi\frac{\ell m}{N}) - \mathrm{Im}(a_m)\sin(2\pi\frac{\ell m}{N})$. The zeroth mode $\ell = 0$, which captures the mean radius, and the first mode $\ell = 1$, which parameterizes translation-like fluctuations, are not relevant in this context.

H. Translational velocity in the Mesh-based Vesicle Model

Simulating the time-evolution of the mesh-based vesicle model for the parameters presented in Table 2 (parameters chosen based on [8]) reveals that the translation velocity observed in the mesh-based simulation is smaller than the velocity expected based the force $\mathbf{F}$ observed in the simulation if we assume that the friction is set by Stokes' law for a vesicle of radius R in a medium with viscosity similar to that of water (Fig. S4). The problem is caused by the choice of the diffusion coefficient of the membrane vertices, D_{mem}, as these coefficients model the viscosity of the medium implicitly. The drag coefficient γ_{mem} for a membrane vertex is set by the vertex diffusion coefficient via the Einstein relation,

$$\gamma_{\mathrm{mem}} = \frac{k_{\mathrm{B}}T}{D_{\mathrm{mem}}}. \tag{S40}$$

Thus, the friction force acting on a single vertex equals $f_i = \gamma_{\mathrm{mem}}\, v = (k_{\mathrm{B}}T)\,/\,(D_{\mathrm{mem}})v$ (for vesicle velocity v), and the total friction acting on all vertices amounts to

$$F_{\mathrm{drag,\,vertex}} = \sum_{i=1}^{N_V} f_i = \frac{k_{\mathrm{B}}T}{D_{\mathrm{mem}}} N_V v \tag{S41}$$

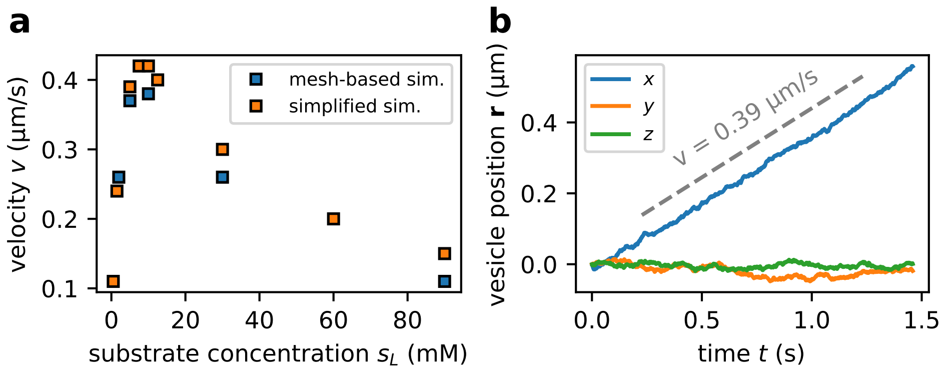

Figure S4. **Translation of enyzme-loaded vesicles**. a) The translation velocities observed in the simplified simulation (blue) and full mesh-based simulation (orange) agree well, provided the viscosity of the medium in the simplified simulation is chosen to match the vertex diffusion coefficient of the mesh-based model. Note that this viscosity $\eta_{\text{used}} \approx 37\,\text{mPa s}$ is about 40 times larger than the true viscosity of water, $\eta_{\text{water}} = 1\,\text{mPa s}$. Every data point shows the translation velocity observed in a single simulated trajectory. b) Time-evolution of the vesicle position (center of mass) in the full mesh-based simulation. The vesicle moves downstream along the substrate gradient with velocity $v \approx 0.39\,\mu\text{m s}^{-1}$ (determined via linear fit). The vesicle is in its high surface-tension state (i.e., no volume constraint). The parameters used for the simulation are listed in Table 2.

Enforcing that this drag force is equal to the drag force expected based on Stokes' law,

$$F_{\text{drag Stokes}} = 6\pi\eta R v, \tag{S42}$$

implies that the membrane vertex diffusion coefficient needs to be chosen as

$$D_{\text{mem}} = \frac{k_B T N_V}{6\pi\eta R}. \tag{S43}$$

The value D_{mem} used in the mesh-based simulation is ≈ 37 times smaller than the desired value according to Eq. (S43) (Table 2 and [8]). However, choosing smaller D_{mem} is challenging due to numerical stability: Using the correct value for D_{mem} requires reducing the integration time step. Albeit this should lead to the response expected for a vesicle in water, the reduction of dt makes the execution of the simulation of a duration in the order of months to years. This long time is a requirement for the equilibration of the enzyme distribution, and therefore this approach is computationally not feasible.

We validated the notion introduced in Eq. (S43) using the simplified simulation: Following Eq. (S43), we set the viscosity in the simplified vesicle model such that it matches the vertex diffusion coefficient used in the full simulation (Table 2). With this choice of viscosity, we find that the translation velocity observed in mesh-based and simplified simulation agree well (Fig. S4).

I. Analysis of Enzyme Distribution

From the simplified simulation, we obtain the three-dimensional positions (x, y, z) of all enzymes in the system as a function time. Using this data, we compute the enzyme profile along the direction of the gradient at a any given time step. To do so, we extract the x-positions of all enzymes, and subtract the vesicle's center of mass (to transform the coordinates to the co-moving frame of the vesicle). We divide the vesicle into spatial bins along its x-direction, and construct a histogram by counting the number of enzymes in each spatial bin. To determine the enzyme concentrations based on the counts, we divide the enzyme count by the volume of the respective bin. Importantly, every bin represents a slice through a spherical vesicle, such that the bin volume differs from one bin to another. Typically, we choose to divide the vesicle into 16 spatial bins, with the left-most bin ending at $x_{\text{left}} = -R - \mathrm{d}R$, and the right-most bin edge at $x_{\text{right}} = R + \mathrm{d}R$. R is the vesicle radius and $\mathrm{d}R$ denotes the average penetration depth, i.e., the typical distance by which enzymes can protrude beyond the vesicle's boundary. (Recall that, in the simplified model, enzymes are confined to the vesicle by a quadratic potential, Eq. (S31)). The total effective volume including the penetration shell

can be estimated from the Boltzmann distribution,

$$V_{\text{eff}} = 4\pi \int_0^\infty \mathrm{d}r\; r^2 \exp\left(-\frac{U_{\text{enz-mem}}(r)}{k_{\mathrm{B}}T}\right) = 4\pi \int_0^R \mathrm{d}r\; r^2 + 4\pi \int_R^\infty \mathrm{d}r\; r^2 \exp\left(-\frac{k_{\text{ves}}(r-R)^2}{k_{\mathrm{B}}T}\right) \tag{S44}$$

$$= \frac{4}{3}\pi R^3 + 2\pi^{3/2} R^2 \lambda + 4\pi R \lambda^2 + \pi^{3/2}\lambda^3 \tag{S45}$$

where we introduced the interface length scale $\lambda = \sqrt{2k_{\mathrm{B}}T/k_{\text{ves}}}$. Based on V_{eff}, we compute the effective radius,

$$R_{\text{eff}} = \sqrt[3]{\frac{3V_{\text{eff}}}{4\pi}} = R\sqrt[3]{1 + \frac{3}{2}\sqrt{\pi}\frac{\lambda}{R} + 3\frac{\lambda^2}{R^2} + \frac{3}{4}\sqrt{\pi}\frac{\lambda^3}{R^3}} \approx R + \frac{\sqrt{\pi}}{2}\lambda$$

We apply this procedure to determine the profile at each time step along the simulation trajectory, excluding the initial relaxation phase (e.g., $t \leq 0.25\,\mathrm{s}$), in which the homogeneous enzyme profile relaxes towards the steady-state profile). We then compute the average enzyme profile and its standard deviation over time. Notably, the bin edges and the number of the bins remain unchanged when evaluating the profile across multiple time steps.

J. Parameters

**Parameters**	**Model Units**	**Physical units**
principal properties		
vesicle radius in equilibrium R	32	$8.0\,\mu\mathrm{m}$
thermal energy unit $k_{\mathrm{B}}T$	0.2	$4.14 \times 10^{-21}\,\mathrm{J}$
time scale τ	1.25×10^5	$7.3\,\mathrm{s}$
Michaelis-Menten-constant K_M	2.8×10^4	$3.0\,\mathrm{mM}$
Vesicle properties		
number of vertices N_V	30000	30000
number of triangles N_T	$2(N_V - 2)$	$2(N_V - 2)$
bending rigidity κ	$20\,k_{\mathrm{B}}T$	8.28×10^{-20} J
average bond length l_b	$4R\sqrt{\pi/(N_T\sqrt{3})}$	$0.176\,\mu\mathrm{m}$
repulsive bond stiffness k_B^{rep}	$2046\,k_{\mathrm{B}}T$	8.47×10^{-18} J/m
attractive bond stiffness k_B^{att}	$10\,k_B^{\text{rep}}$	8.47×10^{-17} J/m
repulsive stiffness λ^{rep}	0.53	0.53
attractive stiffness λ^{att}	0.28	0.28
minimum bond length $l_{\min}$	$0.6\,l_b$	$0.11\,\mu\mathrm{m}$
potential cutoff length l_{c_1}	$0.8\,l_b$	$0.14\,\mu\mathrm{m}$
potential cutoff length l_{c_0}	$1.2\,l_b$	$0.21\,\mu\mathrm{m}$
maximum bond length $l_{\max}$	$1.4\,l_b$	$0.25\,\mu\mathrm{m}$
desired vesicle area A	$4\pi R^2$	$8.04 \times 10^2\,\mu\mathrm{m}^2$
desired vesicle volume V_0	$4\pi R^3/3$	$2.14 \times 10^3\,\mu\mathrm{m}^3$
local area stiffness k_s	$6.43 \times 10^6\,k_{\mathrm{B}}T/A$	3.3×10^{-5} J/m^2
volume stiffness k_V (hyperosmotic condition)	$1.6 \times 10^7\,k_{\mathrm{B}}T/R^3$	129 J/m^3
target volume $\hat{V}$ (hyperosmotic condition)	$0.9V_0$	$1.93 \times 10^3\,\mu\mathrm{m}^3$
volume stiffness k_V (hypoosmotic condition)	$0\,k_{\mathrm{B}}T/R^3$	0 J/m^3
membrane diffusion coefficient D_{m}	$2.5\,R^2/\tau$	$21.9\,\mu\mathrm{m}^2\,\mathrm{s}^{-1}$
flipping frequency ω	$6.4 \times 10^6/\tau$	$8.8 \times 10^5\,\mathrm{s}^{-1}$
flipping probability ψ	0.3	0.3
enzyme properties		
number of enzymes N_e	131,072	$101.5\,\mathrm{nM} \sim 100\,\mathrm{nM}$
number of enzymes N_e in Fig. 2 and S3 under hyperosmotic conditions	55,296	$50.5\,\mathrm{nM} \sim 50\,\mathrm{nM}$
equilibrium diffusion coefficient D_0	3.6×10^{-2}	$39\,\mu\mathrm{m}^2\,\mathrm{s}^{-1}$
enhanced diffusion factor α	0.5	0.5
enz-mem interaction radius σ_{enz}	$1.5\,l_b$	$0.26\,\mu\mathrm{m}$
enz-mem interaction energy param. ϵ	$40\,k_{\mathrm{B}}T$	1.66×10^{-19} J
environmental properties		
maximum substrate concentration $s_{\max}$	$3.33\,K_M$	$10.0\,\mathrm{mM}$
system length L	$2.4\,R$	$19.2\,\mu\mathrm{m}$
further parameters		

time step size dt	2.5×10^{-5}	1.46 ns
total simulation time	2.5×10^{4}	1.46 s

Table 2: Default parameter set used in mesh-based simulations. Principal properties and vesicle properties are the same as in [8] except for a subset of the parameters associated with the bond potential. In individual runs, some of these parameters were varied. In these cases, the affected parameters are stated explicitly, and the remaining independent parameters remain unchanged. The equilibrium enzyme diffusion coefficient and the Michaelis-Menten constant K_{M} are chosen based on the values measured for urease by Jee et al. [29].

**Parameters**	**Model Units**	**Physical units**
principal properties		
vesicle radius R	32	8.0 µm
thermal energy unit $k_{\mathrm{B}}T$	0.2	4.14×10^{-21} J
Michaelis-Menten-constant K_M	2.8×10^{4}	3.0 mM
vesicle properties		
vesicle friction γ_{ves}	$6\pi\eta R$	1.5×10^{-7} Ns/m
vesicle diffusion coefficient D_{ves}	$\frac{k_{\mathrm{B}}T}{\gamma_{\mathrm{ves}}}$	2.8×10^{-2} µm^2 s^{-1}
shell stiffness k_{ves}	$50\, k_{\mathrm{B}}T$	3.3×10^{-6} J/m^2
shell stiffness k_{ves} in Fig. S4	$5\, k_{\mathrm{B}}T$	3.3×10^{-7} J/m^2
shell stiffness k_{ves} for static vesicle in Fig. S6	$5\, k_{\mathrm{B}}T$	3.3×10^{-7} J/m^2
enzyme properties		
number of enzymes N_e	131072	101.5 nM $\sim$ 100 nM
equilibrium diffusion coefficient D_0	3.6×10^{-2}	39 µm^2 s^{-1}
enhanced diffusion factor α	0.5	0.5
environmental properties		
maximum substrate concentration $s_{\max}$	$3.33\, K_M$	10 mM
maximum substrate concentration $s_{\max}$ in Fig. S6	$1.66\, K_M$	5 mM
system length L	76.8	19.2 µm
viscosity of water η	12.9	1.0×10^{-3} Pa s
effective viscosity η in Fig. S4	477.3	37×10^{-3} Pa s
high viscosity η in Figs. S7, S9, S11, S13	12900	1.0 Pa s
general parameters		
time step size dt	0.0051	300 ns
total simulation time	2.5×10^{4}	1.5 s

Table 3. Default parameter set used in simplified simulations. The equilibrium diffusion coefficient and the Michaelis-Menten constant K_M are the values measured for urease by Jee et al. [29].

III. FLUCTUATION SPECTRUM OF ENZYME-FILLED VESICLES

We aim to compare the fluctuation spectrum observed in the simulation to the fluctuation spectrum predicted by theory [8]. To this end, we need to determine the surface tension of vesicle based on the membrane parameters used in the model. Excluding the volume potential, the membrane energy in our system is given by

$$E_m = E_b + E_s, \tag{S46}$$

where E_b is the bending term and E_s is the area stretching term. The bending potential is given by part of the Helfrich Hamiltonian,

$$E_b = 2\kappa \iint H^2 dS\,, \tag{S47}$$

where κ is the bending stiffness and H is the mean curvature. For spherical vesicles, $E_b = 8\pi\kappa$. The area stretching energy equals

$$E_s = \frac{k_s}{2} \sum_{t=1}^{N_t} \frac{(A_t - A_0)^2}{A_0}, \tag{S48}$$

where N_t is the total number of triangles of the mesh, A_t is the current triangle area and A_0 is the characteristic rest area of the triangles. If we consider a spherical shell discretized with triangles of equal area, the expression can be simplified to

$$E_s = 2\pi k_s \frac{\left(R^2 - R_0^2\right)^2}{R_0^2}. \tag{S49}$$

This expression is equivalent to the expected stretching energy cost of a continuum material.

The force due to the membrane potential in the absence of enzymes equals $f_m = f_s + f_b$. We find the force along the radial direction by computing the radial component of the gradient of E_m,

$$f_m = -\partial_R E_m = \frac{8\pi R k_s \left(R_0^2 - R^2\right)}{R_0^2}. \tag{S50}$$

It is worth noting that the bending term does not contribute to the total force on the radial direction since $\partial_R E_b = 0$. In addition to the forces due to the membrane potential, we also need to account for the force exerted by enzymes. The osmotic pressure associated with the enzymes is given by the ideal gas law, $\Pi = k_B T \left(\frac{N_e}{V}\right)$, where N_e is the number of enzymes. Since the pressure equals force per area, $\Pi = f/S$, we can determine the force exerted by the enzymes in a spherical vesicle ($S = 4\pi R^2$, $V = 4/3\pi R^3$),

$$f_{\text{enz}} = \frac{3 n_e k_B T}{R}. \tag{S51}$$

In equilibrium, the forces are balanced, i.e., the force due to the membrane potential and the force exerted by the enzymes need to add up to zero, $f_m(R^*) + f_{\text{enz}}(R^*) = 0$. This condition sets the equilibrium vesicle radius,

$$R^* = R_0 \sqrt{\frac{1 + \sqrt{1 + \frac{3 N_e k_{\text{B}} T}{2\pi R_0^2 k_s}}}{2}}. \tag{S52}$$

To determine the surface tension σ, we resort to the Young-Laplace equation. For an isotropically pressurized spherical shell, the pressure in the shell is related to the surface tension via $\Pi = 2\sigma/R$. Recalling that the only internal force of the membrane is associated with the area stretching (since $f_b = 0$) allows us to express the pressure as a function of f_s, $\Pi = f_s/(4\pi R^2)$. Consequently, the surface tension equals,

$$\sigma = \left.\frac{f_s(R)}{8\pi R}\right|_{R=R^*} = k_s \left(1 - \frac{(R^*)^2}{R_0^2}\right) = \frac{k_s - k_s\sqrt{1 + \frac{3 N_e k_{\text{B}} T}{2\pi R_0^2 k_s}}}{2} \approx -0.97\,\mu\text{N}\,\text{m}^{-1}. \tag{S53}$$

Having determined the surface tension σ and the equilibrium radius R^*, we can compute the resulting equilibrium fluctuation spectrum [8],

$$\left\langle |a_\ell|^2 \right\rangle = \frac{k_B T}{4\pi\sigma} \left(\frac{1}{\ell} - \frac{1}{\sqrt{\frac{\sigma}{\kappa} R^2 + \ell^2}}\right), \tag{S54}$$

where the average radius is $R = R^*$. The fluctuation spectrum approaches $\langle |a_\ell^2| \rangle \sim \ell^{-1}$ in the so-called tension-dominated regime ($\sigma\kappa^{-1} \gg_\ell^2 R^{-2}$), and $\langle |a_\ell^2| \rangle \sim \ell^{-3}$ in the bending-dominated regime ($\sigma\kappa^{-1} \ll \ell^2 R^{-2}$). If the enzymes within the vesicle exhibit no enhanced diffusion ($\alpha = 0$), we find that the fluctuation spectrum obtained in simulations and theoretically predicted equilibrium fluctuation spectrum agree well (Fig. 3). For enzymes displaying enhanced diffusion, large wavelength fluctuations (low mode numbers) have higher amplitudes than in equilibrium. Reducing the tension (area stretching stiffness k_s) of the membrane can increase the amplitude of these deformations even further, but the relative strength of the deformations (i.e., amplitude with enhanced diffusion compared to equilibrium amplitude) should be unaffected by k_s. Note that changes in the bending modulus κ do not affect the large-wavelength deformations caused by enhanced diffusion, as κ only governs the behavior of the fluctuation spectrum for high mode numbers.

IV. TRANSLATIONAL VELOCITY OF ENZYME-FILLED VESICLES

In order to compute the translational velocity of an enzyme-filled vesicle, we need to compute (i) the steady-state concentration profile of the enzymes in the vesicle, as well as (ii) the resulting force acting on the vesicle and the associated velocity. However, the steady-state enzyme profile depends on the translation velocity of the vesicle, implying that it is necessary to know the velocity of the vesicle in order to compute the velocity via the steady-state enzyme profile. We use this notion to formulate a self-consistency condtion for the velocity of the vesicle. Together with an expansion in small Péclet number, the self-consistency approach allows us to derive a closed expression for the vesicle velocity.

A. Steady-State Enyzme Profile

For the derivation of the steady-state enzyme profile, we start from the enhanced diffusion equation that accounts for the motion of the enzyme (Eq. (4)). We assume that the enzyme concentration only depends on the position along the direction of the substrate, which simplifies Eq. (4) in steady-state to

$$\partial_t e = 0 = \partial_x^2 (D_e(x) e(x)) + v\, \partial_x e(x). \tag{S55}$$

The solution of this differential equation reads

$$e(x) = \frac{e(-R) D_e(-R)}{D_e(x)} \exp\left[-v \int_{-R}^{x} \mathrm{d}x' \frac{1}{D_e(x')} \right]. \tag{S56}$$

As we aim to express the enzyme profile as a function of the total concentration, we need to relate $e(-R)$ to the total number of enzymes,

$$N_\mathrm{T} = \int \mathrm{d}V e_\mathrm{T} = \frac{4\pi R^3}{3} e_\mathrm{T} \tag{S57}$$

$$N_\mathrm{T} = \int \mathrm{d}V e(x) = e(-R) D_e(-R) \int_{-R}^{R} \mathrm{d}x \frac{\pi (R^2 - x^2)}{D_e(x)} \exp\left[-v \int_{-R}^{x} \mathrm{d}x' \frac{1}{D_e(x')} \right] \tag{S58}$$

To simplify further, we rescale x in units of radius, i.e., $\tilde{x} = \frac{x}{R}$,

$$N_\mathrm{T} = e(-R) D_e(-R) \pi R^3 \int_{-1}^{1} \mathrm{d}\tilde{x}\, \frac{1 - \tilde{x}^2}{D_e(\tilde{x})} \exp\left[-v \int_{-1}^{\tilde{x}} \mathrm{d}\tilde{x}' \frac{R}{D_e(\tilde{x}')} \right] \tag{S59}$$

Solving for $e(-R)$, we find

$$e(-R) = \frac{\frac{4}{3}\pi R^3 e_\mathrm{T}}{D_e(-R) \pi R^3 \int_{-1}^{1} \mathrm{d}\tilde{x} \frac{1 - x^2}{D_e(\tilde{x})} \exp\left[-v \int_{-1}^{\tilde{x}} \mathrm{d}\tilde{x}' \frac{R}{D_e(\tilde{x}')} \right]} \tag{S60}$$

$$= \frac{4 e_\mathrm{T}}{3 D_e(-R) \int_{-1}^{1} \mathrm{d}\tilde{x} \frac{1 - x^2}{D_e(\tilde{x})} \exp\left[-v \int_{-1}^{\tilde{x}} \mathrm{d}\tilde{x}' \frac{R}{D_e(\tilde{x}')} \right]} \tag{S61}$$

We can plug this back into the formula for the enzyme profile $e(x)$,

$$e(x) = \frac{4 e_\mathrm{T} \frac{1}{D_e(x)} \exp\left[-v \int_{-R}^{x} \mathrm{d}x' \frac{1}{D_e(x')} \right]}{3 \int_{-1}^{1} \mathrm{d}\tilde{x} \frac{1 - x^2}{D_e(\tilde{x})} \exp\left[-v \int_{-1}^{\tilde{x}} \mathrm{d}\tilde{x}' \frac{R}{D_e(\tilde{x}')} \right]}. \tag{S62}$$

For consistency, we also rescale the position x to the dimensionless position $\tilde{x}$ in the numerator,

$$e(\tilde{x}) = \frac{4}{3} e_\mathrm{T} \frac{\frac{1}{D_e(\tilde{x})} \exp\left[-v \int_{-1}^{\tilde{x}} \mathrm{d}\tilde{x}'\, \frac{1}{D_e(\tilde{x}')} \right]}{\int_{-1}^{1} \mathrm{d}\tilde{x} \frac{1 - \tilde{x}^2}{D_e(\tilde{x})} \exp\left[-v \int_{-1}^{\tilde{x}} \mathrm{d}\tilde{x}' \frac{R}{D_e(\tilde{x}')} \right]}. \tag{S63}$$

To make the representation of the enzyme profile fully dimensionless, we introduce the dimensionless enzyme concentration, $\tilde{e}(\tilde{x}) = e(\tilde{x})/e_{\mathrm{T}}$, as well as the dimensionless diffusion coefficient, $\tilde{D}_e(\tilde{x}) = D_e(\tilde{x})/D_{\mathrm{e}}^0$,

$$\tilde{e}(\tilde{x}) = \frac{4}{3}\frac{\frac{1}{\tilde{D}_e(\tilde{x})}\exp\left[-\frac{vR}{D_e^0}\int_{-1}^{\tilde{x}}\mathrm{d}\tilde{x}'\frac{1}{\tilde{D}_e(\tilde{x})}\right]}{\int_{-1}^{1}\mathrm{d}\tilde{x}\frac{1-\tilde{x}^2}{\tilde{D}_e(\tilde{x})}\exp\left[-\frac{vR}{D_e^0}\int_{-1}^{\tilde{x}}\mathrm{d}\tilde{x}'\frac{1}{\tilde{D}_e(\tilde{x}')}\right]} \tag{S64}$$

The dimensionless representation allows us to identify the Péclet number, $\mathrm{Pe} = vR/D_{\mathrm{e}}^0$. Even for the highest velocities observed in the simulation ($v \approx 0.6\,\mathrm{\mu m\,s^{-1}}$ for a vesicle with radius $R = 8\,\mathrm{\mu m}$ and diffusion coefficient $D_{\mathrm{e}}^0 = 39\,\mathrm{\mu m^2\,s^{-1}}$), the Péclet number is small, $\mathrm{Pe} \approx 0.12$, which allows us to expand the enzyme profile, $\tilde{e}(\tilde{x})$ in the Péclet number.
To zeroth order, i.e., taking the limit $\mathrm{Pe} \to 0$, we find the adiabatic enzyme profile,

$$\tilde{e}^{(0)}(\tilde{x}) = \frac{4}{3}\frac{\frac{1}{\tilde{D}_e(\tilde{x})}}{\int_{-1}^{1}\mathrm{d}\tilde{x}\,\frac{1-\tilde{x}^2}{\tilde{D}_e(\tilde{x})}}. \tag{S65}$$

Note that this profile is the solution to the enhanced diffusion equation that does not account for the vesicle drift (Eq. (1)).

To obtain the enzyme profile to linear order in Pe, we expand the exponential functions in small arguments,

$$\tilde{e}(\tilde{x}) \approx \frac{4}{3}\frac{\frac{1}{\tilde{D}_e(\tilde{x})}\left[1 - \mathrm{Pe}\int_{-1}^{\tilde{x}}\frac{1}{\tilde{D}_e(\tilde{x}')}\mathrm{d}\tilde{x}'\right]}{\int_{-1}^{1}\mathrm{d}\tilde{x}\,\frac{1-\tilde{x}^2}{\tilde{D}_e(\tilde{x})}\left[1 - \mathrm{Pe}\int_{-1}^{\tilde{x}}\mathrm{d}\tilde{x}'\frac{1}{\tilde{D}_e(\tilde{x})}\right]} \tag{S66}$$

$$= \frac{4}{3}\frac{\frac{1}{\tilde{D}_e(\tilde{x})}}{\int_{-1}^{1}\mathrm{d}\tilde{x}\,\frac{1-\tilde{x}^2}{\tilde{D}_e(\tilde{x})}\left[1 - \mathrm{Pe}\int_{-1}^{\tilde{x}}\mathrm{d}\tilde{x}'\frac{1}{\tilde{D}_e(\tilde{x})}\right]} - \frac{4}{3}\mathrm{Pe}\frac{\frac{1}{\tilde{D}_e(\tilde{x})}\int_{-1}^{\tilde{x}}\mathrm{d}\tilde{x}'\frac{1}{\tilde{D}_e(\tilde{x}')}}{\int_{-1}^{1}\mathrm{d}\tilde{x}\,\frac{1-\tilde{x}^2}{\tilde{D}_e(\tilde{x})}\left[1 - \mathrm{Pe}\int_{-1}^{\tilde{x}}\mathrm{d}\tilde{x}'\frac{1}{\tilde{D}_e(\tilde{x})}\right]} \tag{S67}$$

$$= \frac{4}{3}\frac{\frac{1}{\tilde{D}_e(\tilde{x})}}{\int_{-1}^{1}\mathrm{d}\tilde{x}\frac{1-\tilde{x}^2}{\tilde{D}_e(\tilde{x})}} + \frac{4}{3}\frac{\frac{1}{\tilde{D}_e(\tilde{x})}}{\int_{-1}^{1}\mathrm{d}\tilde{x}\,\frac{1-\tilde{x}^2}{\tilde{D}_e(\tilde{x})}}\mathrm{Pe}\frac{\int_{-1}^{1}\mathrm{d}\tilde{x}\,\frac{1-\tilde{x}^2}{\tilde{D}_e(\tilde{x})}\int_{-1}^{\tilde{x}}\mathrm{d}\tilde{x}'\frac{1}{\tilde{D}_e(\tilde{x}')}}{\int_{-1}^{1}\mathrm{d}\tilde{x}\frac{1-\tilde{x}^2}{\tilde{D}_e(\tilde{x})}} \tag{S68}$$

$$- \frac{4}{3}\frac{\frac{1}{\tilde{D}_e(\tilde{x})}}{\int_{-1}^{1}\mathrm{d}\tilde{x}\,\frac{1-\tilde{x}^2}{\tilde{D}_e(\tilde{x})}}\mathrm{Pe}\int_{-1}^{\tilde{x}}\mathrm{d}\tilde{x}'\frac{1}{\tilde{D}_e(\tilde{x}')} + \mathcal{O}\left((\mathrm{Pe})^2\right) \tag{S69}$$

$$= \tilde{e}^{(0)}(\tilde{x}) + \mathrm{Pe}\,\tilde{e}^{(0)}(\tilde{x})\,\mathcal{W}^a - \mathrm{Pe}\,\tilde{e}^{(0)}(\tilde{x})\int_{-1}^{\tilde{x}}\mathrm{d}\tilde{x}'\,\frac{1}{\tilde{D}_e(\tilde{x})} + \mathcal{O}\left((\mathrm{Pe})^2\right), \tag{S70}$$

where we introduced a Péclet-number independent weight $\mathcal{W}^a$,

$$\mathcal{W}^a = \frac{\int_{-1}^{1}\mathrm{d}\tilde{x}\,\frac{1-\tilde{x}^2}{\tilde{D}_e(\tilde{x})}\int_{-1}^{\tilde{x}}\mathrm{d}\tilde{x}'\frac{1}{\tilde{D}_e(\tilde{x}')}}{\int_{-1}^{1}\mathrm{d}\tilde{x}\frac{1-\tilde{x}^2}{\tilde{D}_e(\tilde{x})}}. \tag{S71}$$

Thus, the enzyme profile to linear order in Péclet number reads

$$\tilde{e}^{(1)}(\tilde{x}) = \tilde{e}^{(0)}(\tilde{x}) + \mathrm{Pe}\,\tilde{e}^{(0)}(\tilde{x})\,\mathcal{W}^a - \mathrm{Pe}\,\tilde{e}^{(0)}(\tilde{x})\int_{-1}^{\tilde{x}}\mathrm{d}\tilde{x}'\,\frac{1}{\tilde{D}_e(\tilde{x})}. \tag{S72}$$

B. Force and Velocity

The pressure created by a given enzyme profile in the vesicle can be computed via the ideal gas law (equivalent to the dilute limit of van't Hoff's law),

$$\Pi(r,t) = k_{\mathrm{B}}T\,e(r,t)\,, \tag{S73}$$

where Π is the pressure and e the enzyme concentration (number of enzymes per volume unit). The force exerted by the enzymes can be obtained by integrating the pressure over the whole membrane surface S,

$$\mathbf{F} = \oint_S \Pi(x)\mathbf{n}\,\mathrm{d}S = \int_V \nabla\Pi\,\mathrm{d}V = \mathbf{e_x}\,k_\mathrm{B}T\int_{-R}^{R}\pi(R^2-x^2)\partial_x e(x)\mathrm{d}x = \mathbf{e_x}2\pi kT\int_{-R}^{R} xe(x)\,\mathrm{d}x\,. \tag{S74}$$

In the second-to-last step, we again assumed that the enzyme profile is isotropic in the directions perpendicular to the direction of the gradient, i.e., isotropic in the y- and z-direction. Equivalently, we can express the force in terms of the dimensionless representation of the enzyme-profile,

$$\mathbf{F} = \mathbf{e_x}\,2\pi k_\mathrm{B}TR^2e_\mathrm{T}\int_{-1}^{1}\mathrm{d}\tilde{x}\;\tilde{x}\tilde{e}(\tilde{x}). \tag{S75}$$

Provided that the vesicle motion is overdamped and that the vesicle's friction is given by Stokes friction, velocity in x direction resulting from the force is given by

$$v = \frac{F}{6\pi\eta R} = \frac{k_\mathrm{B}Te_\mathrm{T}R}{3\eta}\int_{-1}^{1}\mathrm{d}\tilde{x}\;\tilde{x}\tilde{e}(\tilde{x}). \tag{S76}$$

For the adiabatic enzyme profile, $\tilde{e}^{(0)}(\tilde{x})$, we obtain the adiabatic velocity,

$$v^{(0)} = \frac{k_\mathrm{B}Te_\mathrm{T}R}{3\eta}\int_{-1}^{1}\mathrm{d}\tilde{x}\;\tilde{x}\tilde{e}^{(0)}(\tilde{x}) \tag{S77}$$

(the integral is evaluated explicitely in Sec. IV E and IV F). For the enzyme profile including contributions to linear order in Péclet number, we find the velocity,

$$\begin{aligned}
v^{(1)} &= \frac{k_\mathrm{B}Te_\mathrm{T}R}{3\eta}\int_{-1}^{1}\mathrm{d}\tilde{x}\;\tilde{x}\tilde{e}^{(1)}(\tilde{x}) && \text{(S78)}\\
&= \underbrace{\frac{kTe_\mathrm{T}R}{3\eta}\int_{-1}^{1}\mathrm{d}\tilde{x}\;\tilde{x}\tilde{e}^{(0)}(\tilde{x})}_{=v^{(0)}} + \mathrm{Pe}\;\mathcal{W}^a\underbrace{\frac{kTe_\mathrm{T}R}{3\eta}\int_{-1}^{1}\mathrm{d}\tilde{x}\;\tilde{x}\tilde{e}^{(0)}(\tilde{x})}_{=v^{(0)}} && \text{(S79)}\\
&\quad - \mathrm{Pe}\;\frac{kTe_\mathrm{T}R}{3\eta}\int_{-1}^{1}\mathrm{d}\tilde{x}\;\tilde{x}\tilde{e}^{(0)}(\tilde{x})\int_{-1}^{\tilde{x}}\mathrm{d}\tilde{x}'\;\frac{1}{\tilde{D}_e(\tilde{x}')} && \text{(S80)}\\
&= v^{(0)} + \mathrm{Pe}\,\mathcal{W}^a v^{(0)} - \mathrm{Pe}\,\frac{kTe_\mathrm{T}R}{3\eta}\,\frac{4}{3}\,\frac{\int_{-1}^{1}\mathrm{d}\tilde{x}\;\tilde{x}\frac{1}{\tilde{D}_e(\tilde{x})}\int_{-1}^{\tilde{x}}\mathrm{d}\tilde{x}'\frac{1}{\tilde{D}_e(\tilde{x}')}}{\int_{-1}^{1}\mathrm{d}\tilde{x}\frac{1-\tilde{x}^2}{\tilde{D}_e(\tilde{x})}} && \text{(S81)}\\
&= v^{(0)} + \mathrm{Pe}\,\mathcal{W}^a v^{(0)} - \mathrm{Pe}\,\mathcal{W}^b v^{(0)}, && \text{(S82)}
\end{aligned}$$

where we defined a new Péclet number independent weight,

$$\begin{aligned}
\mathcal{W}^b &= \frac{1}{v^{(0)}}\frac{4}{3}\frac{kTe_\mathrm{tot}R}{3\eta}\,\frac{\int_{-1}^{1}\mathrm{d}\tilde{x}\;\frac{\tilde{x}}{\tilde{D}_e(\tilde{x})}\int_{-1}^{\tilde{x}}\mathrm{d}\tilde{x}'\frac{1}{\tilde{D}_e(\tilde{x}')}}{\int_{-1}^{1}\mathrm{d}\tilde{x}\frac{1-\tilde{x}^2}{\tilde{D}_e(\tilde{x})}} && \text{(S83)}\\
&= \frac{3\eta}{kTe_\mathrm{tot}R}\,\frac{\int_{-1}^{1}\mathrm{d}\tilde{x}\;\frac{1-\tilde{x}^2}{\tilde{D}_e(\tilde{x})}}{\int_{-1}^{1}\mathrm{d}\tilde{x}\frac{\tilde{x}}{\tilde{D}_e(\tilde{x})}}\,\frac{kTe_\mathrm{tot}R}{3\eta}\,\frac{\int_{-1}^{1}\mathrm{d}\tilde{x}\;\frac{\tilde{x}}{\tilde{D}_e(\tilde{x})}\int_{-1}^{\tilde{x}}\mathrm{d}\tilde{x}'\frac{1}{\tilde{D}_e(\tilde{x}')}}{\int_{-1}^{1}\mathrm{d}\tilde{x}\frac{1-\tilde{x}^2}{\tilde{D}_e(\tilde{x})}} && \text{(S84)}\\
&= \frac{\int_{-1}^{1}\mathrm{d}\tilde{x}\;\frac{\tilde{x}}{\tilde{D}_e(\tilde{x})}\int_{-1}^{\tilde{x}}\mathrm{d}\tilde{x}'\frac{1}{\tilde{D}_e(\tilde{x}')}}{\int_{-1}^{1}\mathrm{d}\tilde{x}\frac{\tilde{x}}{\tilde{D}_e(\tilde{x})}}\,. && \text{(S85)}
\end{aligned}$$

Summarizing the weight functions $\mathcal{W}^a$ and $\mathcal{W}^b$ into a single weight, $\mathcal{W} = \mathcal{W}^b - \mathcal{W}^a$, allows us to write the velocity as

$$v^{(1)} = v^{(0)} - \mathrm{Pe}\,\mathcal{W}v^{(0)} = v^{(0)} - \frac{vR}{D_\mathrm{e}^0}\,\mathcal{W}v^{(0)}, \tag{S86}$$

where we used the definition of Pe in the last step. Imposing a self-consistency constraint, i.e., assuming that the translation velocity, v, is well-approximated by the velocity obtained from the expansion to linear order in Péclet number, $v^{(1)}$, allows us to solve for $v = v^{(1)}$,

$$v^{(1)} = \frac{v^{(0)}}{1 + \mathcal{W}\frac{v^{(0)}R}{D_\mathrm{e}^0}}. \tag{S87}$$

In the limit of small adiabatic Péclet number, $\mathrm{Pe}^{(0)} = v^{(0)}R/D_\mathrm{e}^0$, the translation velocity to linear order in Péclet number is well-approximated by the adiabatic velocity,

$$v^{(1)} \approx v^{(0)} \quad \text{if} \quad \mathcal{W}\mathrm{Pe}^{(0)} \ll 1. \tag{S88}$$

For large adiabatic velocity (large adiabatic Péclet number), the $v^{(1)}$ is independent of the adiabatic velocity, and only depends on $\mathcal{W}$,

$$v^{(1)} \approx \frac{D_\mathrm{e}^0}{R\mathcal{W}} \quad \text{if} \quad \mathcal{W}\mathrm{Pe}^{(0)} \gg 1. \tag{S89}$$

C. Effective Trajectory-Averaged Velocity

The translation velocity v depends on the position of the vesicle in the system: As the vesicle moves, the substrate concentration within the vesicle changes, which affects the effective diffusion coefficient of the enzymes, and, consequently, the pressure and the ensuing translation velocity. In the derivation so far, we assumed that the center of the vesicle is placed in the middle of the system, $x = 0$, such that the vesicle "sees" the part of the substrate gradient between $x = -R$ and $x = R$ (the whole system extends from $x = -L/2$ to $x = L/2$). As the vesicle moves, the position of the center of the vesicle x_V changes and the relevant part of the substrate gradient is between $= x_V - R$ and $x = x_V + R$. We need to account for this by shifting the position at which the substrate gradient (and thus the effective diffusion coefficient) is evaluated, $x \to x + x_V$ (i.e., $\tilde{x} \to \tilde{x} + x_V/R$). With this shift, the full steady-state enzyme profile reads (analogous to Eq. (S64))

$$\tilde{e}(\tilde{x}, x_V) = \frac{4}{3}\frac{\frac{1}{\tilde{D}_e(\tilde{x}+x_V/R)}\exp\left[-\frac{vR}{D_\mathrm{e}^0}\int_{-1}^{\tilde{x}}\mathrm{d}\tilde{x}'\frac{1}{\tilde{D}_e(\tilde{x}'+x_V/R)}\right]}{\int_{-1}^{1}\mathrm{d}\tilde{x}\,\frac{1-\tilde{x}^2}{\tilde{D}_e(\tilde{x}+x_V/R)}\exp\left[-\frac{vR}{D_\mathrm{e}^0}\int_{-1}^{\tilde{x}}\mathrm{d}\tilde{x}'\frac{1}{\tilde{D}_e(\tilde{x}'+x_V/R)}\right]}. \tag{S90}$$

Consequently, the velocity of the vesicle,

$$v(x_V) = \frac{k_\mathrm{B}Te_\mathrm{T}R}{3\eta}\int_{-1}^{1}\mathrm{d}\tilde{x}\,\tilde{x}\,\tilde{e}(\tilde{x}, x_V), \tag{S91}$$

depends on the position of the vesicle and varies along its trajectory. The velocity observed in the simulation is the effective vesicle velocity v^eff averaged over the positions of the vesicle along its trajectory. To compute this effective velocity, we need to determine the distance by which the vesicle moves, $\Delta x(t) = x(t) - x(0)$, by integrating the equation of motion,

$$\frac{\mathrm{d}x_V(t)}{\mathrm{d}t} = v^{(1)}(x_V). \tag{S92}$$

Provided the change in the position of the vesicle is small enough, we can expand the velocity to linear order in x,

$$\frac{\mathrm{d}x_V(t)}{\mathrm{d}t} \approx v^{(1)}(x_V = 0) + \left.\frac{\partial v^{(1)}(x_V)}{\partial x_V}\right|_{x_V=0} x_V. \tag{S93}$$

This differential equation can be solved analytically,

$$x_V(t) - x_V(0) = \frac{v^{(1)}(x_V = 0)}{\left.\frac{\partial v^{(1)}}{\partial x_V}\right|_{x_V=0}}\left[\exp\left(\left.\frac{\partial v}{\partial x_V}\right|_{x_V=0} t\right) - 1\right], \tag{S94}$$

leading to the following expression for the effective (trajectory-averaged) velocity,

$$v^{\text{eff}}(t) = \frac{x_V(t) - x_V(0)}{t} = \frac{1}{t}\frac{v^{(1)}(x_V = 0)}{\frac{\partial v^{(1)}}{\partial x_V}\big|_{x_V=0}}\left[\exp\left(\frac{\partial v}{\partial x_V}\bigg|_{x_V=0} t\right) - 1\right]. \tag{S95}$$

The velocity at position $x = 0$ to linear order in Péclet number, $v^{(1)}(x = 0)$, is computed via Eq. (S87). To evaluate $\partial_{x_V} v^{(1)}(x_V)|_{x_V=0}$, we make use of autodifferentiation. The theoretically predicted effective velocities are plotted in Fig. 5 as continuous curves, and agree well with the translation velocities observed in the simulation.

We note that the vesicle trajectory often appears to be linear, suggesting that a constant translation velocity with negligible position dependence might suffice to explain the observed trajectory, for example, in Fig. 4a. However, when we compute the trajectory by integrating a fixed velocity, $v(x_V = 0)$ (chosen as the vesicle's velocity at its initial position, $x_V = 0$), we observe a significant deviation from the simulation data (Fig. S5a, green curve). In contrast, incorporating the position-dependent velocity, $v(x_V)$, allows the theoretical model to accurately capture the simulation behavior (Fig. S5a, orange curve). While the vesicle reaches the position $x_{\text{pos. dep. vel.}} = 0.94\,\mu\text{m}$ after $1.5\,\text{s}$ accounting for the position-dependent velocity, the distance travelled during the same time equals $x_{\text{const. vel.}} = 0.87\,\mu\text{m}$ assuming constant velocity. This implies that the effective velocity $v^{\text{eff}} = \frac{x_{\text{pos. dep. vel.}}}{t} = 0.63\,\mu\text{m}\,\text{s}^{-1}$ deviates by about 9% from the initial velocity, $v(x_V = 0) = 0.58\,\mu\text{m}\,\text{s}^{-1}$. This difference arises because the velocity varies appreciably along the trajectory (Fig. S5b), even though the resulting non-linearity is subtle and not immediately apparent in Fig. 4a.

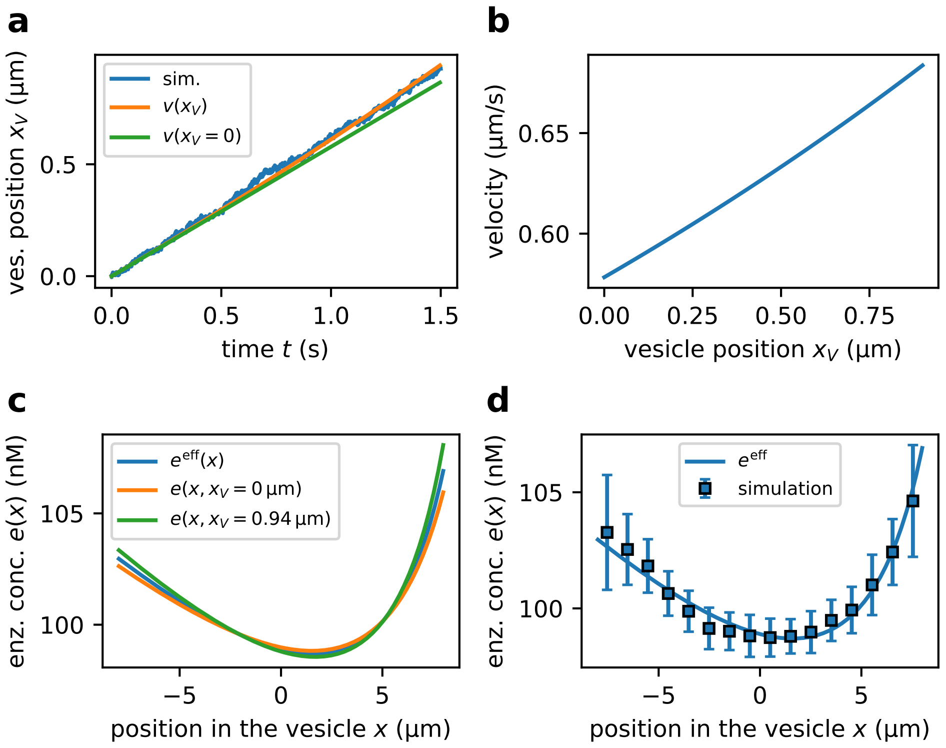

Figure S5. **Position-dependence of translation velocity and enzyme profile.** a) Time-evolution of the vesicle position observed in the simplified simulation compared to the trajectory obtained assuming a position-independent velocity, $v(x_V = 0\,\mu\text{m})$, and position-dependent velocity $v(x_V)$. b) The velocity depends on the position of the vesicle in the system, with higher velocity reached further to the right. c) The enzyme profile within the vesicle depends on the position of the vesicle in the system, with steeper enzyme profiles further right in the system. d) The trajectory-averaged enzyme profile agrees well with the average enzyme profile observed in the simulations (average and standard deviation computed along trajectory including data for $t \geq 0.25\,\text{s}$). System parameters are summarized in Table 3.

D. Effective Trajectory-Averaged Enzyme Profile

Just like the velocity of the vesicle, the enzyme profile also depends on the position of the vesicle within the system (Eq. S90, and Fig. S5c). Therefore, the average enzyme profile observed in the simulation corresponds to the effective enzyme profile $e^{\text{eff}}(x)$ averaged over the positions of the vesicle along its trajectory,

$$e^{\text{eff}}(x) = \frac{1}{T}\int_0^T \mathrm{d}t\; e(x, x_V(t))\,, \tag{S96}$$

where $x_V(t)$ denotes the time-dependent position of vesicle in the system, while x denotes the position within the vesicle, i.e., in the co-moving frame of the vesicle. The integral with respect to t can be expressed as an integral with respect to the position of the vesicle,

$$e^{\text{eff}}(x) = \frac{1}{T}\int_0^{x_V(T)} \mathrm{d}x_V\; \frac{e(x, x_V)}{v^{(1)}(x_V) + \frac{\partial v^{(1)}(x_V)}{\partial x_V}\Big|_{x_V=0} x_V}\,, \tag{S97}$$

We can interpret this expression as a weighted average of enzyme profiles, with the inverse velocity of the vesicle as the weighting factor. We compute $e^{\text{eff}}(x)$ by evaluating the integral in Eq. (S97) numerically, and find that the effective enzyme profile agrees well with the enzyme profiles obtained in the simulation (Fig. 4b, Fig. S5d).

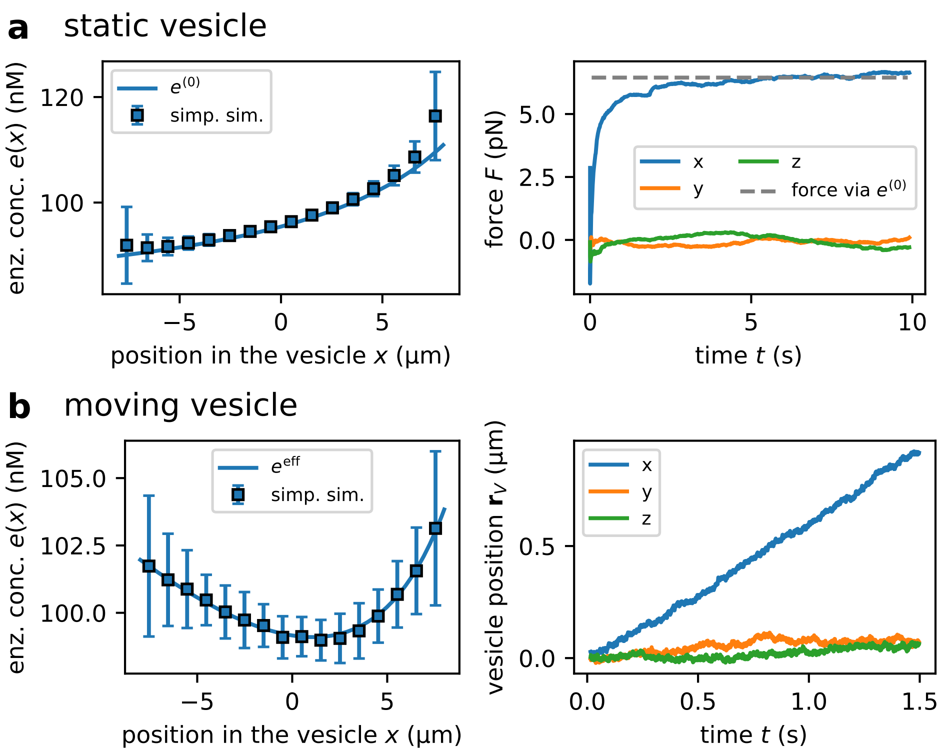

Figure S6. **Comparison of static and moving vesicles.** a) The steady-state enzyme profile observed in the simulation of a static vesicle agrees well with the adiabatic enzyme profile obtained analytically (left panel). Here, the steady-state enzyme profile and its standard deviation is determined from the simulation data by averaging the enzyme profiles over time, including data for $t \geq 4\,\text{s}$. Likewise, the steady-state force exerted by the enzymes agrees well with the theoretically expected force (right panel). b) The trajectory-averaged enzyme profile observed in the simulation of the moving vesicle agrees well with the analytical effective trajectory-averaged enzyme profile, e^{eff}, but shows considerable fluctuations around its average value (left panel). The average profile is obtained from the simulation data by averaging enzyme profiles over time, including data for $t \geq 0.25\,\text{s}$. The vesicle moves to the right along the direction of the gradient (right panel). The system parameters are summarized in Table 3.

E. Substrate-Dependence of the Velocity

In this section we consider the substrate-dependence of the velocity. We assume that the substrate concentration equals zero on the right end of the system, while it attains a variable non-zero concentration s_ℓ on the left end of the

system. As discussed before (see Section IV B), $v^{(1)}$ is well-approximated by the adiabatic velocity $v^{(0)}$ in the limit of small adiabatic Péclet number (i.e., $\mathcal{W}\,\mathrm{Pe}^{(0)} \ll 1$). Therefore, we need to determine the substrate-dependence of $v^{(0)}$ in order to understand the scaling of $v^{(1)}$ with the substrate in this limit.
Combining the definition of the adiabatic velocity (Eq. (S77)) with the adiabatic enzyme profile (Eq. (S65)), we find the following expression for the adiabatic velocity,

$$v^{(0)} = \frac{4k_{\mathrm{B}}Te_{\mathrm{T}}R}{9\eta} \frac{\int_{-1}^{1} \mathrm{d}\tilde{x}\, \frac{\tilde{x}}{\tilde{D}_e(\tilde{x})}}{\int_{-1}^{1} \mathrm{d}\tilde{x}\, \frac{1-\tilde{x}^2}{\tilde{D}_e(\tilde{x})}} = \frac{4k_{\mathrm{B}}Te_{\mathrm{T}}R}{9\eta} \frac{\mathcal{I}_1}{\mathcal{I}_2}, \tag{S98}$$

where we introduced the integrals,

$$\mathcal{I}_1 = \int_{-1}^{1} \mathrm{d}\tilde{x}\, \frac{\tilde{x}}{\tilde{D}_e(\tilde{x})}, \quad \text{and} \quad \mathcal{I}_2 = \int_{-1}^{1} \mathrm{d}\tilde{x}\, \frac{1-\tilde{x}^2}{\tilde{D}_e(\tilde{x})}. \tag{S99}$$

We recall that the dimensionless diffusion coefficient is a function of the position-dependent substrate concentration (Eq. (2)),

$$\tilde{D}_e(\tilde{x}) = 1 + \alpha \frac{s(\tilde{x})}{K_{\mathrm{M}} + s(\tilde{x})}, \tag{S100}$$

with the substrate concentration,

$$s(\tilde{x}) = \frac{s_\ell}{2} - \frac{s_\ell}{L} R\tilde{x}. \tag{S101}$$

As mentioned before, s_ℓ denotes the concentration on the left end of the system, and the concentration on the right end of the system is assumed to be zero. The linear position-dependence of the substrate allows us express the position $\tilde{x}$ as function of the concentration,

$$\tilde{x} = \frac{L}{2R} - \frac{L}{Rs_\ell} s \tag{S102}$$

such that we can re-parametrize the integrals with respect to $\tilde{x}$ (Eq. (S99)) as integrals with respect to s,

$$\mathcal{I}_1 = \int_{-1}^{1} \mathrm{d}\tilde{x}\, \frac{\tilde{x}}{\tilde{D}_e(\tilde{x})} = -\frac{L}{s_\ell R} \int_{\frac{s_\ell}{2} - \frac{s_\ell R}{L}}^{\frac{s_\ell}{2} + \frac{s_\ell R}{L}} \mathrm{d}s\, \frac{\frac{L}{2R} - \frac{L}{Rs_\ell} s}{\tilde{D}_e(s)}, \tag{S103}$$

$$\mathcal{I}_2 = \int_{-1}^{1} \mathrm{d}\tilde{x}\, \frac{1-\tilde{x}^2}{\tilde{D}_e(\tilde{x})} = -\frac{L}{s_\ell R} \int_{\frac{s_\ell}{2} - \frac{s_\ell R}{L}}^{\frac{s_\ell}{2} + \frac{s_\ell R}{L}} \mathrm{d}s\, \frac{1 - \left(\frac{L}{2R} - \frac{L}{Rs_\ell} s\right)^2}{\tilde{D}_e(s)}. \tag{S104}$$

Unlike the integrals with respect to $\tilde{x}$, these integrals with respect to s can be evaluated using a computer algebra system. To rationalize the scaling of $v^{(0)}$ with the substrate concentration at the left-end of the system, s_ℓ, we use the expressions for the integrals $\mathcal{I}_1$ and $\mathcal{I}_2$ obtained via the computer algebra system to compute $v^{(0)}$, and expand $1/v^{(0)}$ in small concentrations,

$$\frac{1}{v^{(0)}} = \frac{9\eta}{4k_{\mathrm{B}}Te_{\mathrm{T}}R} \left(\frac{2K_{\mathrm{M}}L}{\alpha R} \frac{1}{s_\ell} + \frac{(2+\alpha)L}{\alpha R} + \frac{(1+\alpha)(5L^2 - 4(3+2\alpha)R^2)}{10(\alpha K_{\mathrm{M}} LR)} s_\ell \right) + \mathcal{O}(s_\ell^2). \tag{S105}$$

This result allows us to identify two regimes: (i) For small substrate concentrations, $1/v^{(0)}$ is proportional to $1/s_\ell$, implying that the velocity scales linearly with $v^{(0)} \sim s_\ell$, and (ii) for large substrate concentration, $1/v^{(0)}$ is proportional to s_ℓ, implying that $v^{(0)} \sim 1/s_\ell$. The characteristic substrate concentration $s_\ell^{*\,(0)}$ that separates the two regimes from each other is set by the condition that both contributions ($\sim s_\ell^{-1}$ and $\sim s_\ell$) need to contribute equally to the velocity,

$$\frac{2K_M L}{\alpha R s_\ell^{*\,(0)}} = \frac{(1+\alpha)(5L^2 - 4(3+2\alpha)R^2) s_\ell^{*\,(0)}}{10(\alpha K_{\mathrm{M}} LR)}, \tag{S106}$$

which leads to the following expression for the characteristic substrate concentration,

$$s_\ell^{*\,a} = \frac{\sqrt{20} K_M L}{\sqrt{(1+\alpha)(5L^2 - 4(3+2\alpha)R^2)}}. \tag{S107}$$

Note that this concentration coincides with the position of the maximum, as can be verified by finding the concentration that solves $\partial_{s_\ell}(v^{(0)})^{-1} = 0$.

In the limit of large adiabatic Péclet number, $\mathcal{W}\,\mathrm{Pe}^{(0)} \gg 1$, $v^{(0)}$ does not approximate $v^{(1)}$ anymore. Instead, the velocity is given by

$$v^{(1)} \approx \frac{D_\mathrm{e}^0}{R\mathcal{W}}. \tag{S108}$$

To understand the substrate-dependence of the translation velocity in this limit, we need to evaluate $\mathcal{W}$ (Eq. (S71) and (S85)),

$$\mathcal{W} = \frac{\int_{-1}^{1} \mathrm{d}\tilde{x}\, \frac{\tilde{x}}{\tilde{D}_e(\tilde{x})} \int_{-1}^{\tilde{x}} \mathrm{d}\tilde{x}' \frac{1}{\tilde{D}_e(\tilde{x}')}}{\int_{-1}^{1} \mathrm{d}\tilde{x} \frac{\tilde{x}}{\tilde{D}_e(\tilde{x})}} - \frac{\int_{-1}^{1} \mathrm{d}\tilde{x}\, \frac{1-\tilde{x}^2}{\tilde{D}_e(\tilde{x})} \int_{-1}^{\tilde{x}} \mathrm{d}\tilde{x}' \frac{1}{\tilde{D}_e(\tilde{x}')}}{\int_{-1}^{1} \mathrm{d}\tilde{x} \frac{1-\tilde{x}^2}{\tilde{D}_e(\tilde{x})}} = \frac{\mathcal{I}_3}{\mathcal{I}_1} - \frac{\mathcal{I}_4}{\mathcal{I}_2}, \tag{S109}$$

where we re-used the integrals $\mathcal{I}_1$ and $\mathcal{I}_2$ introduced previously, and defined,

$$\mathcal{I}_0(\tilde{x}) = \int_{-1}^{\tilde{x}} \mathrm{d}\tilde{x}' \frac{1}{\tilde{D}_e(\tilde{x}')}, \quad \mathcal{I}_3 = \int_{-1}^{1} \mathrm{d}\tilde{x}\, \frac{\tilde{x}}{\tilde{D}_e(\tilde{x})} \mathcal{I}_0(\tilde{x}), \quad \mathcal{I}_4 = \int_{-1}^{1} \mathrm{d}\tilde{x}\, \frac{1-\tilde{x}^2}{\tilde{D}_e(\tilde{x})} \mathcal{I}_0(\tilde{x}) \tag{S110}$$

Expressing the dimensionless position $\tilde{x}$ in terms of the substrate concentration s (Eq. (S102)), allows us to identify the following representation of the integrals,

$$\mathcal{I}_0(s) = -\frac{L}{s_\ell R} \int_{\frac{s_\ell}{2} - \frac{s_\ell R}{L}}^{s} \mathrm{d}s' \frac{1}{\tilde{D}_e(s)}, \tag{S111}$$

$$\mathcal{I}_3 = -\frac{L}{s_\ell R} \int_{\frac{s_\ell}{2} - \frac{s_\ell R}{L}}^{\frac{s_\ell}{2} + \frac{s_\ell R}{L}} \mathrm{d}s\, \frac{\mathcal{I}_0(s) \left(\frac{L}{2R} - \frac{L}{R s_\ell} s \right)}{\tilde{D}_e(s)}, \tag{S112}$$

$$\mathcal{I}_4 = -\frac{L}{s_\ell R} \int_{\frac{s_\ell}{2} - \frac{s_\ell R}{L}}^{\frac{s_\ell}{2} + \frac{s_\ell R}{L}} \mathrm{d}s\, \frac{\mathcal{I}_0(s) \left[1 - \left(\frac{L}{2R} - \frac{L}{R s_\ell} s \right)^2 \right]}{\tilde{D}_e(s)}. \tag{S113}$$

Using a computer algebra system to evaluate these integrals allows us to find an analytical expression for $\mathcal{W}$. Expanding in small s_ℓ, we obtain the following expression for $\mathcal{W}$,

$$\mathcal{W} = \frac{K_\mathrm{M} L}{\alpha R} \frac{1}{s_\ell} + \frac{L}{\alpha R} + \frac{5L^2 - 4(3 + 2\alpha - \alpha^2) R^2}{20 \alpha K_\mathrm{M} L R} s_\ell + \mathcal{O}(s_\ell^2). \tag{S114}$$

Just as previously, this implies that there are two regimes: (i) For small s_ℓ, the first term in Eq. (S114) dominates, such that $\mathcal{W} \sim s_\ell^{-1}$. As the velocity is inversely proportional to $\mathcal{W}$, the velocity scales linearly in substrate concentration, $v^{(1)} \sim s_\ell$ for small substrate concentrations. (ii) For large s_ℓ, the third term in Eq. (S114) is contributes most significantly, such that the velocity decays as $v^{(1)} \sim s_\ell^{-1}$. We can again identify the characteristic substrate concentration for the transition between the regimes (i.e., the concentration at which both terms contribute equally to the velocity),

$$s_\ell^{*\,b} = \frac{\sqrt{20} K_M L}{\sqrt{5L^2 - 4(3 + 2\alpha - \alpha^2) R^2}} \tag{S115}$$

Note that this characteristic substrate concentration $s_\ell^{*\,b}$ can differ from the characteristic concentration obtained for the adiabatic velocity, $s_\ell^{*\,a}$. In summary, we find that

$$v^{(1)} \sim \begin{cases} s_\ell^{-1} & \text{if} \quad \mathcal{W}\mathrm{Pe}^{(0)} \ll 1 \quad \text{and} \quad s_\ell \ll s_\ell^{*\,a}, \\ s_\ell & \text{if} \quad \mathcal{W}\mathrm{Pe}^{(0)} \ll 1 \quad \text{and} \quad s_\ell \gg s_\ell^{*\,a}, \\ s_\ell^{-1} & \text{if} \quad \mathcal{W}\mathrm{Pe}^{(0)} \gg 1 \quad \text{and} \quad s_\ell \ll s_\ell^{*\,b}, \\ s_\ell & \text{if} \quad \mathcal{W}\mathrm{Pe}^{(0)} \gg 1 \quad \text{and} \quad s_\ell \gg s_\ell^{*\,b}. \end{cases} \tag{S116}$$

For a vesicle moving in a medium of high viscosity (i.e., $\eta = 1\,\mathrm{Pa\,s}$), the velocity of translation is well-approximated by the adiabatic velocity $v^{(0)}$ over the entire range of studied substrate concentrations (Fig. S7A-B). Thus, the first two

cases in Eq. (S116) apply: The velocity reaches a maximum at $s_\ell^{*\,a}$. For concentrations smaller than $s_\ell^{*\,a}$, the velocity increases linearly with s_ℓ, while it decreases linearly with s_ℓ^{-1} for concentrations larger than $s_\ell^{*\,a}$ (Fig. S7C). Fig. S7 shows the velocity of a vesicle placed at $x = 0$ (no trajectory averaging!), the trajectory-averaged effective velocity is plotted in Fig. S13A. Given the small velocities (and consequently small displacement), the effecitve velocity and the velocity at $x = 0$ are almost identical.

If the vesicle moves in a medium of water-like viscosity (i.e., $\eta = 1\,\mathrm{mPa\,s}$), the velocity of translation is well-approximated by the $D_\mathrm{e}^0\,/\,(\mathcal{W}R)$ over the entire range of studied substrate concentrations (Fig. S8A-B). Thus, the last two cases in Eq. (S116) apply: The velocity reaches a maximum at $s_\ell^{*\,b}$. For concentrations smaller than $s_\ell^{*\,b}$, the velocity increases linearly with s_ℓ, while it decreases linearly with s_ℓ^{-1} for concentrations larger than $s_\ell^{*\,b}$ (Fig. S8C). Again, Fig. S8 depicts the translation velocity for a vesicle at $x = 0$, the respective effective velocity is plotted in Fig. 5A.

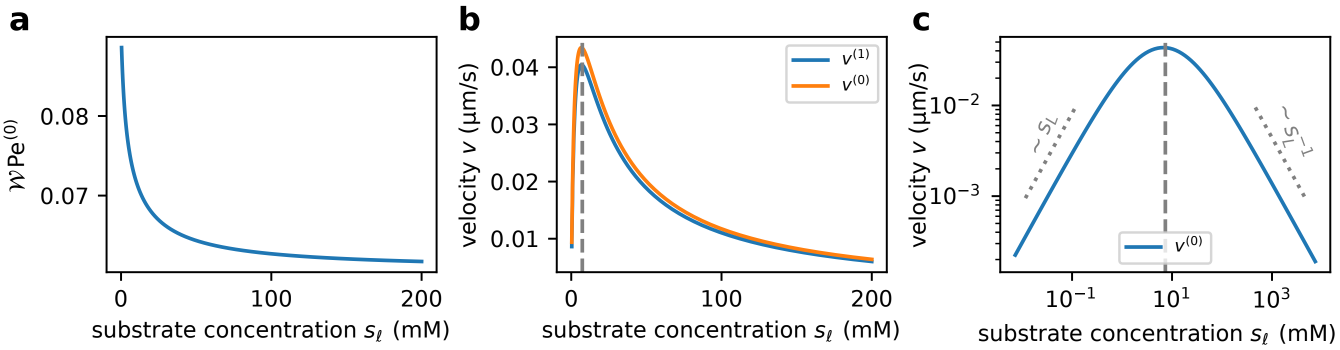

Figure S7. **Substrate-dependence of the translation velocity for high viscosity, $\eta = 1\,\mathrm{Pa\,s}$ (without trajectory averaging of the velocity)**. a) The order parameter $\mathcal{W}\mathrm{Pe}^{(0)}$ is small over the entire studied range of parameters, implying that the translation velocity $v^{(1)}$ is well-approximated by the adiabatic velocity $v^{(0)}$ (see panel B). b) The translation velocity exhibits a non-monotoneous dependence on the substrate concentration, reaching a maximum at intermediary substrate concentration, $s_\ell = s_\ell^{*\,a}$ (see grey vertical dashed line). c) For small substrate concentration, $v^{(0)}$ is proportional to s_ℓ, while for large substrate concentration $v^{(0)} \sim s_\ell^{-1}$ (see power laws plotted as dashed lines). In all panels, the velocities are computed using the system parameters summarized in Table 3.

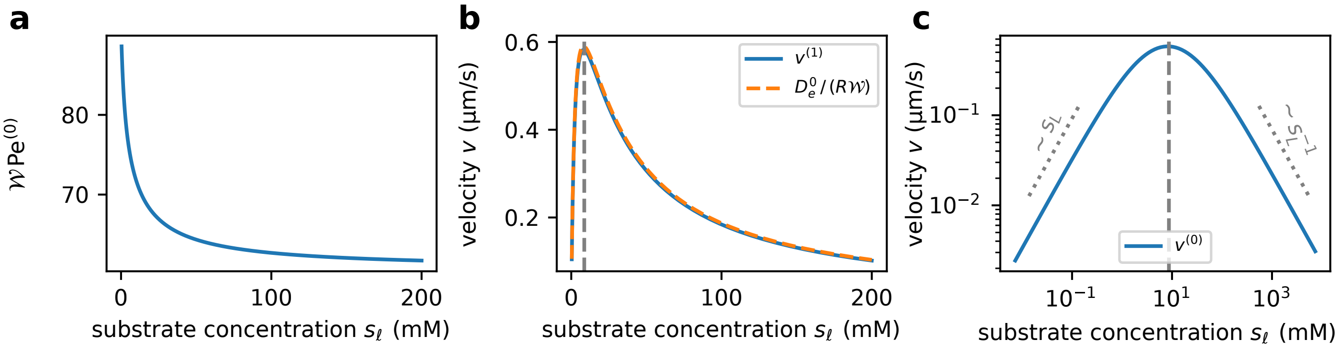

Figure S8. **Substrate-dependence of the translation velocity for small viscosity, $\eta = 1\,\mathrm{mPa\,s}$ (without trajectory averaging of the velocity).** a) The order parameter $\mathcal{W}\mathrm{Pe}^{(0)}$ is much larger than 1 over the entire studied range of parameters, implying that the translation velocity $v^{(1)}$ is well-approximated by the $D_\mathrm{e}^0\,/\,(R\mathcal{W})$ (see panel B). b) The translation velocity exhibits a non-monotoneous dependence on the substrate concentration, reaching a maximum at intermediary substrate concentration, $s_\ell = s_\ell^{*\,b}$ (see grey vertical dashed line). c) For small substrate concentration, $v^{(0)}$ is proportional to s_ℓ, while for large substrate concentration $v^{(0)} \sim s_\ell^{-1}$ (see power laws plotted as dashed lines). All parameters (except for the viscosity) are identical to the parameters summarized in Table 3.

F. Radius-Dependence of the Velocity

Similarly as in the last section, we analyze the radius-dependence of $v^{(1)}$ in the limit of small and high adiabatic Péclet number separately. In the limit of small adiabatic Péclet number, $v^{(1)}$ is well-approximated by $v^{(0)}$. We start by analyzing the radius-dependence of $v^{(0)}$: We use the representation of the adiabatic velocity in terms of the integrals $\mathcal{I}_1$ and $\mathcal{I}_2$ introduced in the last section (Eqs. (S98) and (S99)), and expand the expression for $v^{(0)}$ (obtained via a computer algebra system) in small vesicle radius R,

$$v^{(0)} = \frac{4k_{\mathrm{B}}Te_{\mathrm{T}}}{9\eta}\frac{2\alpha K_{\mathrm{M}}s_\ell R^2}{L(2K_{\mathrm{M}}+s_\ell)(2K_{\mathrm{M}}+(1+\alpha)s_\ell)} + \mathcal{O}(R^3). \tag{S117}$$

Hence, in the limit $\mathcal{W}\mathrm{Pe}^{(0)} \ll 1$, $v^{(1)}$ depends quadratically on the vesicle radius, in the same way as $v^{(0)}$.
However, in the limit $\mathcal{W}\,\mathrm{Pe}^{(0)} \gg 1$, the velocity is given by $v^{(1)} \approx D_{\mathrm{e}}^0\,/\,(R\mathcal{W})$. To understand the radius dependence of the translation velocity in this limit, we express $\mathcal{W}$ in terms of the integrals $\mathcal{I}_0(s)$, $\mathcal{I}_3$ and $\mathcal{I}_4$ introduced previously (Eq. (S110)). Expanding $\mathcal{W}$ in small R, we obtain the following expression for $\mathcal{W}$,

$$\mathcal{W} \approx \mathcal{W}_{-1}R^{-1} + \mathcal{W}_1 R + \mathcal{W}_3 R^3, \tag{S118}$$

with radius-independent constants,

$$\mathcal{W}_{-1} = \frac{L(2K_M+s_\ell)^2}{4\alpha K_M s_\ell}, \tag{S119}$$

$$\mathcal{W}_1 = \frac{(1+\alpha)\ s_\ell\ (2K_M+s_\ell)\ [2(\alpha-3)K_M - 3(1+\alpha)s_\ell]}{5\alpha k_M L(2K_M+(1+\alpha)s_\ell)^2}. \tag{S120}$$

This representation of $\mathcal{W}$ implies the following radius-dependence for the velocity,

$$v^{(1)} \approx \frac{D_{\mathrm{e}}^0}{\mathcal{W}_{-1} + \mathcal{W}_1 R^2 + \mathcal{W}_3 R^4}. \tag{S121}$$

In addition to the scaling of $v^{(1)}$ for large radii, the expression for $\mathcal{W}$ (Eq. (S118)) also allows us to derive an analytical expression for the characteristic radius up to which the velocity $v^{(1)}$ is well-approximated by the adiabatic velocity. To this end, we use the criterion,

$$\mathcal{W}\,\mathrm{Pe}^{(0)} = \frac{\mathcal{W}(R^*)v^{(0)}(R^*)R^*}{D_{\mathrm{e}}^0} = 1, \tag{S122}$$

as well as the radius-dependent expression for $\mathcal{W} \approx \mathcal{W}_{-1}/R$ and $v^{(0)}$ (Eq. (S117)),

$$\frac{1}{D_{\mathrm{e}}^0}\frac{L(2K_{\mathrm{M}}+s_\ell)^2}{4\alpha K_{\mathrm{M}}s_\ell}\frac{4k_{\mathrm{B}}Te_{\mathrm{T}}}{9\eta}\frac{2\alpha K_{\mathrm{M}}s_\ell (R^*)^2}{L(2K_{\mathrm{M}}+s_\ell)(2K_{\mathrm{M}}+(1+\alpha)s_\ell)} = 1 \tag{S123}$$

$$\frac{2K_{\mathrm{M}}+s_\ell}{D_{\mathrm{e}}^0}\frac{2k_{\mathrm{B}}Te_{\mathrm{T}}}{9\eta}\frac{(R^*)^2}{(2K_{\mathrm{M}}+(1+\alpha)s_\ell)} = 1, \tag{S124}$$

which leads to the following expression for the characteristic radius,

$$R^* = \sqrt{\frac{9\eta D_{\mathrm{e}}^0\,(2K_{\mathrm{M}}+(1+\alpha)s_\ell)}{2k_{\mathrm{B}}Te_{\mathrm{T}}\,(2K_{\mathrm{M}}+s_\ell)}}. \tag{S125}$$

In summary, we find,

$$v^{(1)} \approx \begin{cases} v^{(0)} & \sim R^2 \qquad\qquad \text{for} \quad R < R^*, \\ \dfrac{D_{\mathrm{e}}^0}{R\mathcal{W}} & \sim (\mathcal{W}_{-1}+\mathcal{W}_1R^2+\mathcal{W}_3R^4)^{-1} \quad \text{for} \quad R > R^*. \end{cases} \tag{S126}$$

For a vesicle moving in a medium of high viscosity (i.e., $\eta = 1\,\mathrm{Pa\,s}$), $R^* \approx 30\,\mu\mathrm{m}$ for the investigated parameters, implying that the velocity scales quadratically with the radius for all studied radii, as shown in Fig. S9 (adiabatic velocity without trajectory-averaging) and Fig. S13 (effective velocity with trajectory-averaging).
If the vesicle moves in a medium of water-like viscosity (i.e., $\eta = 1\,\mathrm{mPa\,s}$), the characteristic radius equals $R^* \approx 1\,\mu\mathrm{m}$. Consequently, we observe both regimes introduced in Eq. (S126), as shown in Fig. S10B or Fig. 5b (see orange curve for $v^{(0)}$ and green curve for $D_{\mathrm{e}}^0/(R\mathcal{W})$ as well as the vertical dashed line representing R^*). Note that the representation in Fig. 5 shows the trajectory-averaged velocity computed based on $v^{(1)}$, while Fig. S10B shows the bare velocity $v^{(1)}$ without trajectory averaging. Moreover, we find that Eq. (S121) is indeed a good approximation of $D_{\mathrm{e}}^0/(R\mathcal{W})$ (Fig. S10C).

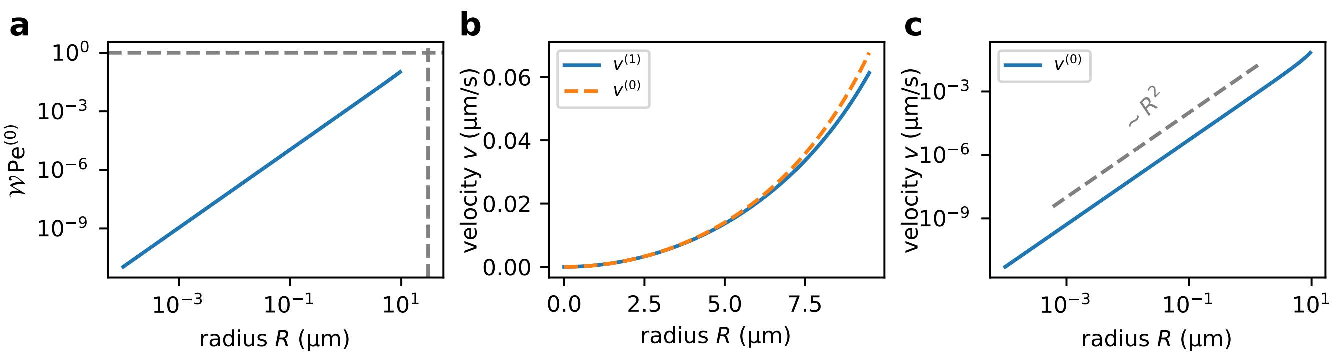

Figure S9. **Radius-dependence of the vesicle velocity for high viscosity, $\eta = 1\,\mathrm{Pa\,s}$ (without trajectory averaging).** a) The order parameter $\mathcal{W}\,\mathrm{Pe}^{(0)}$ is smaller than 1 over the entire range of studied radii. The characteristic radius R^* (see vertical dashed line) is larger than the largest radius to be analyzed. b) Over the entire range of studied radii, $v^{(1)}$ is well-approximated by $v^{(0)}$. c) The adiabatic velocity $v^{(0)}$ (and thus $v^{(1)}$) scales quadratically with the vesicle radius R. All parameters are identical to the ones listed in Table 3 with a fixed substrate concentration of $s_\ell = 10\,\mathrm{mM}$.

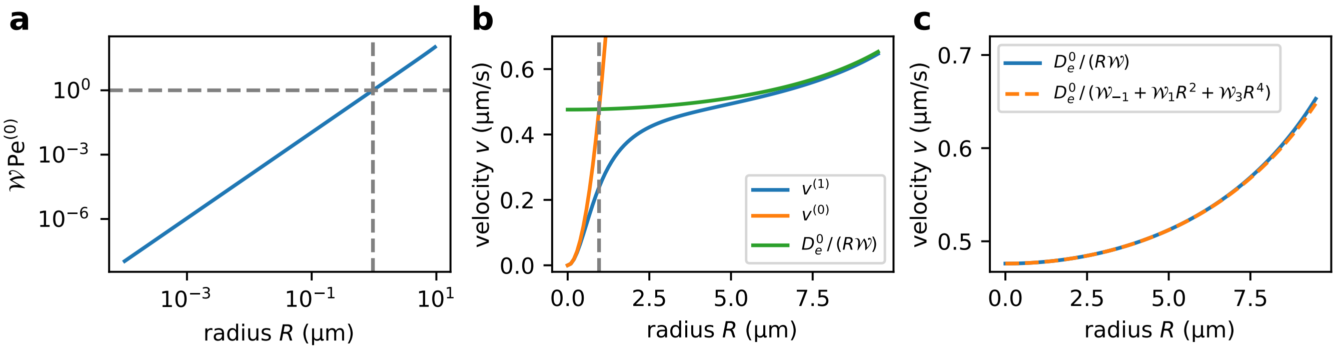

Figure S10. **Radius-dependence of the vesicle velocity for small viscosity, $\eta = 1\,\mathrm{mPa\,s}$ (without trajectory averaging).** a) The order parameter $\mathcal{W}\,\mathrm{Pe}^{(0)}$ is smaller than 1 for $R < R^*$, and larger than 1 for $R > R^*$. The characteristic radius R^* (vertical dashed line) is set by the condition $\mathcal{W}\,\mathrm{Pe}^{(0)} = 1$ (horizontal dashed line). b) For small radii, $R < R^*$, $v^{(1)}$ is well-approximated by $v^{(0)}$, while $v^{(1)} \approx D_\mathrm{e}^0/(R\mathcal{W})$ for large radii, $R > R^*$. The characteristic radius R^* is shown as vertical dashed line. c) $D_\mathrm{e}^0/(\mathcal{W}_{-1} + \mathcal{W}_1 R + \mathcal{W}_3 R^4)$ is a good approximation for $D_\mathrm{e}^0/(R\mathcal{W})$ over the entire studied range of radii. All parameters (except for the viscosity) are identical to the ones listed in Table 3.

G. Dependence of the Velocity on the Diffusion Coefficient

To understand the dependence of the translation velocity $v^{(1)}$ on the diffusion coefficient, we analyze how the adiabatic velocity $v^{(0)}$ and $D_\mathrm{e}^0\,/\,(R\mathcal{W})$ depend on D_e^0:

- The adiabatic velocity $v^{(0)}$ is independent of D_e^0, since the integrals $\mathcal{I}_1$ and $I_\in$ appearing in $v^{(0)}$ (Eq. (S98)) only depend on the *dimensionless* diffusion coeffcient $\tilde{D}_e(\tilde{x})$ (and therefore not on D_e^0).
- $D_\mathrm{e}^0\,/\,(R\mathcal{W})$ depends linearly on D_e^0, as the integrals appearing in $\mathcal{W}$ (Eq. (S109)) are independent of D_e^0.

In summary, we find,

$$v^{(1)} \sim \begin{cases} 1 & \text{if} \quad \mathcal{W}\mathrm{Pe}^{(0)} \ll 1, \\ D_\mathrm{e}^0 & \text{if} \quad \mathcal{W}\mathrm{Pe}^{(0)} \gg 1. \end{cases} \tag{S127}$$

For a vesicle that moves in a medium of high viscosity (i.e., $\eta = 1\,\mathrm{Pa\,s}$), the translation velocity $v^{(1)}$ is set by $D_\mathrm{e}^0\,/\,(\mathcal{W}R)$ as long as the diffusion coefficient is small, such that the velocity increases linearly with D_e^0 (see green dashed line in Fig. S11b). As the diffusion coefficient D_e^0 increases, the dimensionless order parameter $\mathcal{W}\,\mathrm{Pe}^{(0)}$ decreases, implying that $v^{(1)}$ is well-approximated by $v^{(0)}$. Consequently, the velocity $v^{(1)}$ approaches the adiabatic velocity $v^{(0)}$ asymptotically, and $v^{(1)}$ is independent of D_e^0 in the limit of high diffusion coefficients.

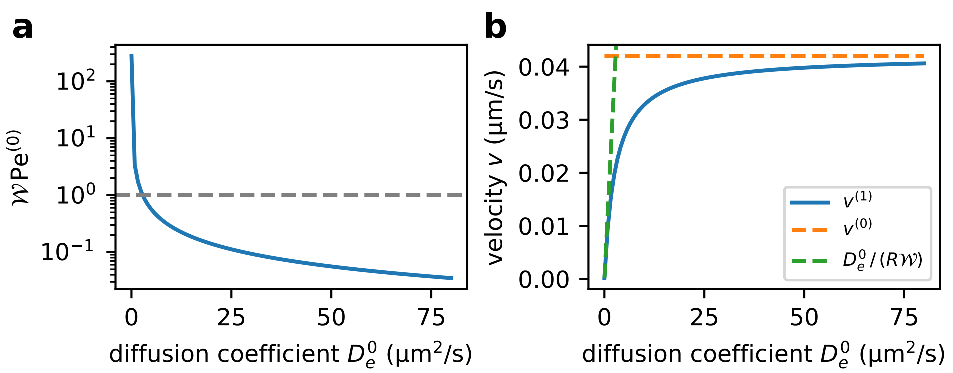

Figure S11. **Dependence of the vesicle velocity on the diffusion coefficient for high viscosity, $\eta = 1\,\mathrm{Pa\,s}$ (without trajectory averaging).** a) The order parameter $\mathcal{W}\,\mathrm{Pe}^{(0)}$ is smaller than 1 for small diffusion coefficient, but falls below 1 for larger D_e^0. b) For small diffusion coefficient, $v^{(1)}$ is proportional to D_e^0 as it is set by $D_\mathrm{e}^0/(R\mathcal{W})$ (green dashed line). For sufficiently large D_e^0, $v^{(1)}$ approaches the D_e^0-independent adiabatic velocity $v^{(0)}$ (orange dashed line). All parameters are identical to the ones listed in Table 3 with a fixed substrate concentration of $s_\ell = 10\,\mathrm{mM}$.

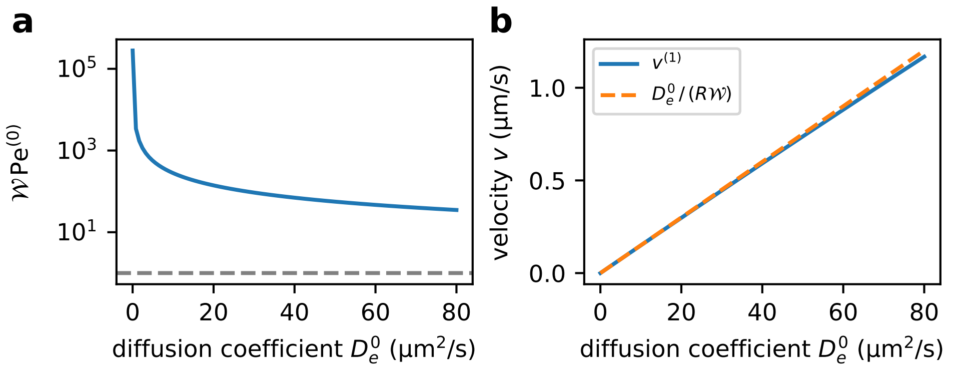

Figure S12. **Dependence of the vesicle velocity on the diffusion coefficient for small viscosity, $\eta = 1\,\mathrm{mPa\,s}$ (without trajectory averaging).** a) The order parameter $\mathcal{W}\,\mathrm{Pe}^{(0)}$ is larger than 1 over the entire range of diffusion coefficients D_e^0. b) The velocity $v^{(1)}$ is proportional to D_e^0 as it is set by $D_\mathrm{e}^0/(R\mathcal{W})$ (green dashed line). All parameters (except for the viscosity) are identical to the parameters listed in Table 3.

V. DYNAMICS AND FLUCTUATIONS OF ENZYME DISTRIBUTION

A. Relaxation of the Initial Enzyme Distribution

A key transient effect in the enzyme distribution is the relaxation of the initial profile (typically uniform in simplified simulations) towards its steady-state distribution. We can study the relaxation dynamics quantitatively by computing the deviation $d(t)$ between the simulated enzyme profile and the corresponding steady-state profile,

$$d(t) = \sqrt{\frac{1}{N_p}\sum_{i=0}^{N_p-1}\left(e_t^{\mathrm{sim}}(x_i) - e_{\mathrm{ss}}^{\mathrm{theo}}(x_i)\right)^2}.$$

were x_i denotes the discrete positions within the vesicle along the direction of the gradient at which the profiles are compared ($N_p = 16$ evenly spaced positions).

By plotting $d(t)$ as a function of time (using the same trajectory as the one presented in Main Text Fig. 4), we find that $d(t)$ decays exponentially. This allows us to determine the relaxation time scale by fitting the autocorrelation function of $d(t)$ to an exponential function, yielding $\tau = 0.07\,\mathrm{s}$ for the plotted trajectory. The steady-state profile observed after relaxation agrees well with the theoretically expected profile (Fig. S14b). While the "steady-state" profile technically evolves as the vesicle moves within the system (Sec. IV D, Fig. S5), this effect is minor, making

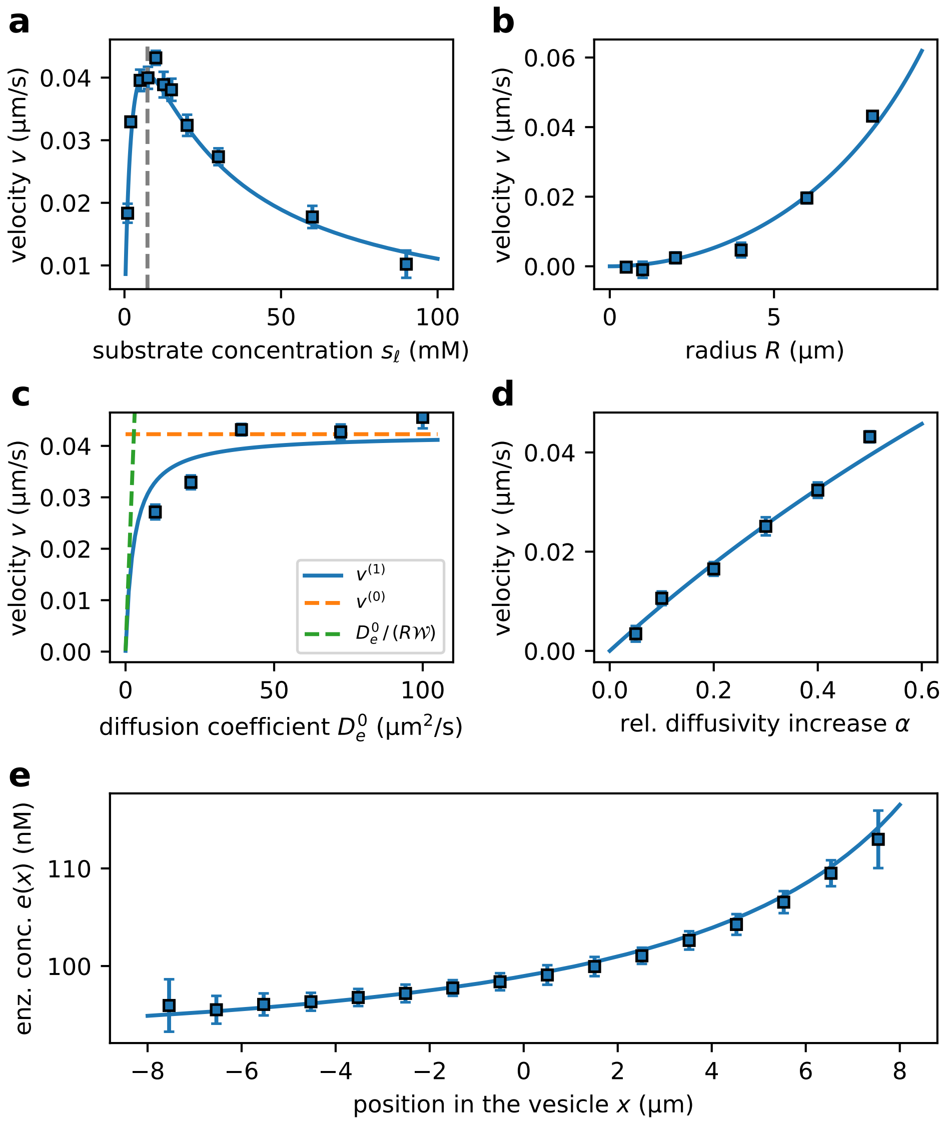

Figure S13. **Parameter-dependence of the vesicle for high viscosity (with trajectory averaging)** a) Vesicle velocity v depends non-monotonously on the substrate concentration on the left edge of the system, s_ℓ. b) Vesicle velocity v increases quadratically as a function of vesicle radius R. c) Vesicle velocity v increases with the diffusion coefficient D_e^0, and approaches the adiabatic velocity $v^{(0)}$ asymptotically in the limit of high D_e^0. d) Vesicle velocity v increases with the strength of enhanced diffusion, α. All data points in the panels a)-d) show the mean velocity and its standard deviation obtained from at least 10 simulation runs (and at most 950 runs for $R = 0.5\,\mu\mathrm{m}$) per data point. e) The steady-state enzyme concentration profile (average and standard deviation computed along a single trajectory including data for $t \geq 0.25\,\mathrm{s}$) agrees well with the analytically predicted effective trajectory averaged enzyme profile e^{eff}. All panels show the effective trajectory-averaged translation velocity in a medium with viscosity that is a factor 1000 higher than that of water, $\eta = 1\,\mathrm{Pa\,s}$. All other parameters are summarized in Table 3

the profile appear effectively constant. During relaxation, the vesicle's effective velocity is slightly lower than after equilibration (Fig. S14c), which is intuitive: A more shallow enzyme profile generates weaker forces compared to the steady-state profile.

We applied this analysis (Fig. S15) to all simulations in the parameter scan (Fig. 5). In all cases, the relaxation timescale is significantly shorter than the simulated trajectory duration and also shorter than $t = 0.25\,\mathrm{s}$, the time we typically exclude from steady-state analyses to avoid biases introduced by the relaxation of the initial enzyme profile.

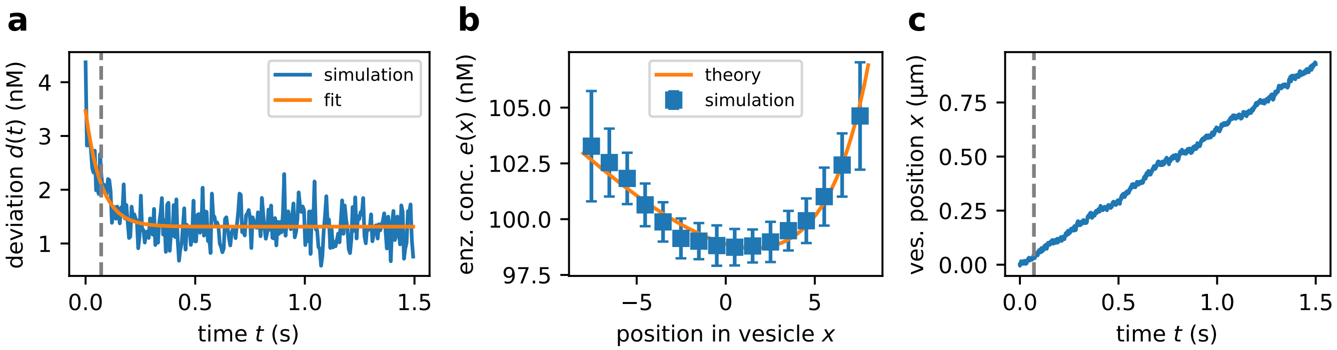

Figure S14. **Relaxation towards steady state.** a) The initially uniform profile relaxes exponentially towards the steady-state profile, timescale $\tau = 0.07\,\mathrm{s}$. b) The trajectory-average of the enzyme profiles emerging in the simulation after relaxation agrees with the theoretically expected steady-state profile. Error bars show the variance of the profile along the simulation. c) The vesicle moves towards the right, with slightly reduced translation velocity during the initial relaxation. Simulation parameters are summarized in Table 3.

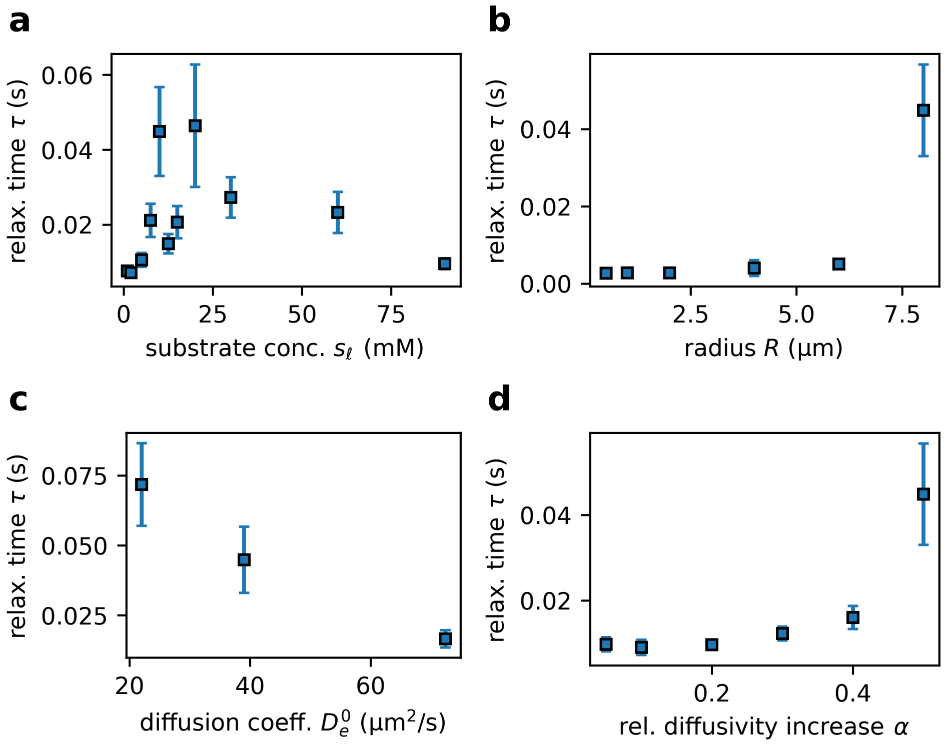

Figure S15. **Influence of system properties on the relaxation timescale.** a) The relaxation time peaks at intermediate substrate concentrations, similar to the translation velocity. At this peak, the enzyme profile is steepest and deviates most from the initial profile, requiring the longest relaxation time. b) and c) Relaxation is diffusion-limited: Increasing vesicle size or decreasing the diffusion coefficient prolongs the relaxation process. d) Higher values of α steepen the enzyme profile, resulting in longer relaxation timescales. The data points show the average relaxation timescales as well as the standard deviation of the mean obtained across at least 10 simulated trajectories. Parameters not varied in this figure are summarized in Table 3.

B. Fluctuations around the Steady-State Enzyme Distribution

Even though the enzyme profile reaches a (quasi-)steady state after the relaxation, fluctuations of the enzyme profile around the steady state are substantial, particularly for smaller vesicles. To study the magnitude of the fluctuations as a function of the vesicle size, we select a single simulation run per vesicle radius and computing the trajectory-averaged

enzyme profile along with its standard deviation over time (for $t \geq 0.25\,\mathrm{s}$),

$$\mu[e(x)] = \frac{1}{N_t}\sum_{i=0}^{N_t-1} e^{\mathrm{sim}}_{t_i}(x), \qquad \sigma[e(x)] = \left(\frac{1}{N_t}\sum_{i=0}^{N_t-1}\left(e^{\mathrm{sim}}_{t_i}(x) - \mu[e(x)]\right)^2\right)^{1/2},$$

where x_i represents discrete positions along the vesicle in the direction of the gradient, and $N_p = 16$ denotes the number of evenly spaced positions. While the average enzyme profile aligns well with the analytically predicted steady-state profile across all vesicle sizes, fluctuations around this mean are considerable (Fig. S16). Note that the magnitude of the fluctuations depends on the position within the vesicle: We determine the enzyme concentration by counting the number of enzymes within slices of the vesicle and dividing by the slice volume. Since enzyme number fluctuations are relatively uniform throughout the vesicle, but the slice volume varies with x, fluctuations in the enzyme profile are smallest at the vesicle center and largest near its edges.

As expected, the magnitude of fluctuations depends on vesicle size: Smaller vesicles (with fewer enzymes) exhibit stronger fluctuations. To quantify this trend, we analyze the relative fluctuation magnitude at the vesicle center, defined as $r = \sigma[e(x=0)]/\mu[e(x=0)]$, as a function of vesicle radius. For each radius, we compute $\mu[e(x=0)]$ and $\sigma[e(x=0)]$ from at least 10 simulation runs (up to 900 for vesicles with $R = 0.5\,\mu\mathrm{m}$), then determine the mean relative fluctuation strength $\bar{r}$ and its uncertainty Δr using Gaussian error propagation,

$$\bar{\mu} = \frac{1}{N_{\mathrm{runs}}}\sum_{i=0}^{N_{\mathrm{runs}}} \mu_i[e(x=0)], \qquad \Delta\mu = \left(\frac{1}{N_{\mathrm{runs}}}\sum_{i=0}^{N_{\mathrm{runs}}}\left(\mu_i[e(x=0)] - \bar{\mu}\right)^2\right)^{1/2}$$

$$\bar{\sigma} = \frac{1}{N_{\mathrm{runs}}}\sum_{i=0}^{N_{\mathrm{runs}}} \sigma_i[e(x=0)], \qquad \Delta\sigma = \left(\frac{1}{N_{\mathrm{runs}}}\sum_{i=0}^{N_{\mathrm{runs}}}\left(\sigma_i[e(x=0)] - \bar{\sigma}\right)^2\right)^{1/2}$$

$$\bar{r} = \frac{\bar{\sigma}}{\bar{\mu}}, \qquad \Delta r = \left(\frac{\Delta\sigma^2}{\bar{\mu}^2} + \frac{\bar{\sigma}^2}{\bar{\mu}^4}\Delta\mu^2\right)^{1/2}$$

In finite-particle systems, relative fluctuation strength $\bar{r}$ is expected to scale as $N^{-1/2}$ [30]. Since enzyme concentration remains constant across simulations, the enzyme number scales with vesicle volume, $N \sim V \sim R^3$, leading to a relative fluctuation scaling of $R^{-3/2}$ (Fig. S17).

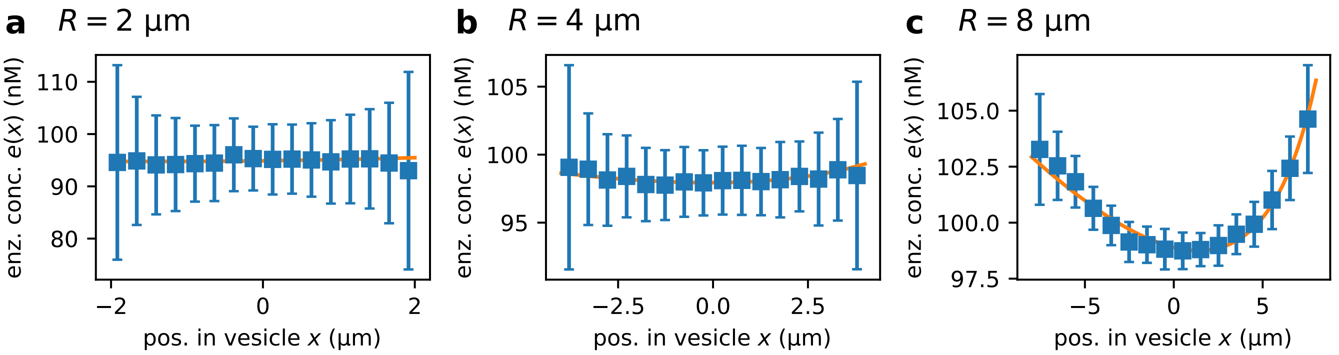

Figure S16. **Enzyme profiles as a function of vesicle radius.** a) $R = 2\,\mu\mathrm{m}$, b) $R = 4\,\mu\mathrm{m}$, and c) $R = 8\,\mu\mathrm{m}$. The theoretically predicted effective trajectory-averaged steady-state enzyme profile (e^{eff}) agrees with the average profiles obtained from the simulation (including data for $t \geq 0.25\,\mathrm{s}$). The fluctuations are larger for smaller vesicles (smaller number of volumes), and their strength depends on the position within the vesicle: We observe larger fluctuations on the ends of the vesicle due to the smaller volume of the slice of the sphere used to determine the concentration. Simulation parameters are summarized in Table 3.

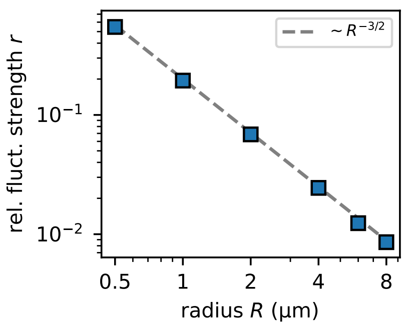

Figure S17. **Fluctuations in enzyme distribution as a function of vesicle radius.** The relative magnitude of fluctuations of enzyme concentration in the middle of the vesicle decreases with $N^{-1/2} \sim R^{-3/2}$ as expected in systems with finite system size. Data points are obtained by averaging over at least 10 ($R = 8\,\mu\mathrm{m}$) and at most 950 ($R = 0.5\,\mu\mathrm{m}$) simulation runs, the uncertainties Δr are smaller than marker size. All simulation parameters are summarized in Table 3.

VI. IDEAS FOR EXPERIMENTAL DESIGNS

A. Substrate Gradient

Commercially available microfluidic cells (e.g., Ibidi μ-slide) allow the generation of substrate gradients in absence of buffer flows [31]. Alternatively, substrate gradients can be generated under constant flow conditions using a tree-like microfluidic network chamber [32], or using Y-shaped chambers where two inlets, each with a given substrate concentration, converge into a main cell under laminar flow conditions [33]. In the latter case, the substrate profiles usually display a sigmoidal shape [34], and measurements can be performed along the linear regime.

B. Vesicles Encapsulating Enzymes

Vesicles encapsulating enzymes can be produced using established techniques, such as double layer emulsion methods or continuous droplet interface crossing encapsulation [4]. Altering the lipid composition of the membrane can change the stiffness of the encapsulating membranes, leading to vesicles with more or less pronounced deformations (Fig. 3 and Sec. III). For example, including cholesterol makes the membrane stiffer [35]. In addition, membrane pores, either based on proteins such as α-Hemolysin or on DNA origami technologies [4, 36], can be used to achieve sufficient permeability to the substrate (Sec. I).

C. Measuring Deformation and Motion

After placing the enzyme-loaded vesicles in the substrate gradient produced via a microfluidic device, its evolution can be tracked using bright field microscopy or confocal microscopy [8]. The microscopy images show 2D cross-sections of the vesicle's shapes, and this information can be used to compute shape observables which are analogous to the shape parameters discussed in this work. For example, in our simulations, we computed the ellipticity based on the cross-section of the vesicle to allow for a simple comparison between experiment and simulation. Subtle deformations can be quantified from the membrane fluctuation spectrum (Fig. 3), which can be determined based on bright-field microscopy or via phase contrast microscopy [28].

Translation velocities, measured by tracking vesicles [37], could be compared to the theoretically predicted velocities, to verify signatures such as the non-monotonous dependency of the velocity on the applied substrate gradient (maximum given by Eq. 8). To determine the dependency of the velocity on the diffusion coefficient, different enzymes displaying enhanced diffusion could be compared [29]. Furthermore, fusion proteins could be used to modify the hydrodynamic radius of the enzymes, and hence the diffusion coefficient.

Using fluorescently labeled enzymes, it might be possible to characterize the density of enzymes along the gradient axis, even though the small differences in enzyme density along the vesicle might pose a challenge. Attaching the vesicle to the surface of the microfluidic cell, which can be achieved by appropriately functionalizing the surface (e.g., antibodies or biotin-streptavidin links), permits to measure the enzyme profile in a non-moving vesicle, which shows a steeper enzyme profile and should be measurable more easily.

Another interesting experimental possibility involves trapping vesicles using optical tweezers. Using the optical trap, the position of the vesicles can be restrained (no motion), and the forces exerted by the enzymes can be quantified. Notably, optical tweezers in combination with fluorescence microscopy [38] would permit to determine the enzyme distribution, forces, as well as translation and/or vesicle deformations.

LIST OF FIGURES

S1 Pore-dependence of membrane permeability . . . 4
S2 Effect of reduced permeability on vesicle propulsion . . . 5
S3 Shape parameters of vesicles in hyper- and hypoosmotic conditions . . . 9
S4 Translation of enzyme-loaded vesicles in full mesh-based simulation . . . 10
S5 Position-dependence of translation velocity and enzyme profile . . . 18
S6 Comparison of static and moving vesicles . . . 19
S7 Substrate-dependence of the translation velocity for high viscosity . . . 22
S8 Substrate-dependence of the translation velocity for small viscosity . . . 22
S9 Radius-dependence of the vesicle velocity for high viscosity . . . 24
S10 Radius-dependence of the vesicle velocity for small viscosity . . . 24
S11 D_e^0-dependence of the vesicle velocity for high viscosity . . . 25
S12 D_e^0-dependence of the vesicle velocity for small viscosity . . . 25
S13 Parameter-dependence of the vesicle velocity for high viscosity . . . 26
S14 Relaxation towards steady state . . . 27
S15 Influence of system properties on the relaxation timescale . . . 27
S16 Enzyme profiles as a function of vesicle radius . . . 28
S17 Fluctuations in enzyme distribution as a function of vesicle radius . . . 29

REFERENCES

[1] R. R. Mayrand and D. G. Levitt, Urea and ethylene glycol-facilitated transport systems in the human red cell membrane. saturation, competition, and asymmetry, J. Gen. Physiol. **81**, 221 (1983).
[2] J. Winkelmann, Diffusion coefficient of urea in water: Datasheet from physical chemistry · volume 15b2: “diffusion in gases, liquids and electrolytes” in springermaterials (2018).
[3] B. Yang, Transport characteristics of urea transporter-b, in *Urea Transporters* (Springer Netherlands, 2014) pp. 127–135.
[4] L. Van de Cauter, F. Fanalista, L. van Buren, N. De Franceschi, E. Godino, S. Bouw, C. Danelon, C. Dekker, G. H. Koenderink, and K. A. Ganzinger, Optimized cdice for efficient reconstitution of biological systems in giant unilamellar vesicles, ACS Synth. Biol. **10**, 1690 (2021).
[5] L. Song, M. R. Hobaugh, C. Shustak, S. Cheley, H. Bayley, and J. E. Gouaux, Structure of staphylococcal alpha-hemolysin, a heptameric transmembrane pore, Science **274**, 1859 (1996).
[6] L. Y. Huang, W. A. Catterall, and G. Ehrenstein, Selectivity of cations and nonelectrolytes for acetylcholine-activated channels in cultured muscle cells., J. Gen. Physiol. **71**, 397 (1978).
[7] D. J. McGillivray, G. Valincius, F. Heinrich, J. W. Robertson, D. J. Vanderah, W. Febo-Ayala, I. Ignatjev, M. Lösche, and J. J. Kasianowicz, Structure of functional staphylococcus aureus α-hemolysin channels in tethered bilayer lipid membranes, Biophysical journal **96**, 1547 (2009).
[8] H. R. Vutukuri, M. Hoore, C. Abaurrea-Velasco, L. van Buren, A. Dutto, T. Auth, D. A. Fedosov, G. Gompper, and J. Vermant, Active particles induce large shape deformations in giant lipid vesicles, Nature **586**, 52 (2020).
[9] N. Kučerka, J. F. Nagle, J. N. Sachs, S. E. Feller, J. Pencer, A. Jackson, and J. Katsaras, Lipid bilayer structure determined by the simultaneous analysis of neutron and x-ray scattering data, Biophys. J. **95**, 2356 (2008).
[10] N. Kucerka, S. Tristram-Nagle, and J. Nagle, Structure of fully hydrated fluid phase lipid bilayers with monounsaturated chains, J. Membr. Biol. **208**, 193 (2006).
[11] N. Kucerka, S. Tristram-Nagle, and J. Nagle, Closer look at structure of fully hydrated fluid phase dppc bilayers, Biophys. J. **90**, L83 (2006).
[12] P. Peterlin, G. Jaklič, and T. Pisanski, Determining membrane permeability of giant phospholipid vesicles from a series of videomicroscopy images, Meas. Sci. Technol. **20**, 055801 (2009).
[13] H. Saito and W. Shinoda, Cholesterol effect on water permeability through dppc and psm lipid bilayers: A molecular dynamics study, J. Phys. Chem. B **115**, 15241 (2011).
[14] W. G. Hill, R. L. Rivers, and M. L. Zeidel, "role of leaflet asymmetry in the permeability of model biological membranes to protons, solutes, and gases, J. Gen. Physiol. **114**, 405 (1999).
[15] M. Palaiokostas, W. Ding, G. Shahane, and M. Orsi, Effects of lipid composition on membrane permeation, Soft Matter **14**, 8496 (2018).
[16] J. J. Foo, K. K. Liu, and V. Chan, Viscous drag of deformed vesicles in optical trap: Experiments and simulations, AIChE Journal **50**, 249–254 (2004).
[17] W. Helfrich, Elastic properties of lipid bilayers: theory and possible experiments, Z. Naturforsch. C. **28**, 693 (1973).
[18] G. Gompper and D. Kroll, Random surface discretizations and the renormalization of the bending rigidity, Journal de Physique I **6**, 1305 (1996).
[19] H. Noguchi and G. Gompper, Dynamics of fluid vesicles in shear flow: Effect of membrane viscosity and thermal fluctuations, Phys. Rev. E **72**, 011901 (2005).

[20] U. Seifert, Configurations of fluid membranes and vesicles, Advances in Physics **46**, 13 (1997).
[21] S.-l. Lien and J. T. Kajiya, A symbolic method for calculating the integral properties of arbitrary nonconvex polyhedra, IEEE Computer Graphics and Applications **4**, 35 (1984).
[22] C. L. Pastrana, L. Qiu, S. Armon, U. Gerland, and A. Amir, Pressure-induced shape-shifting of helical bacteria, Soft Matter **19**, 2224 (2023).
[23] D. H. Boal and M. Rao, Topology changes in fluid membranes, Phys. Rev. A **46**, 3037 (1992).
[24] D. Cohen-Steiner and F. Da, A greedy delaunay based surface reconstruction algorithm, Visual Comput. **20**, 4 (2004).
[25] T. K. F. Da and D. Cohen-Steiner, Advancing front surface reconstruction, The Computational Geometry Algorithms Library (CGAL) User and Reference Manual (2023).
[26] J. Aronovitz and D. Nelson, Universal features of polymer shapes, Journal de physique **47**, 1445 (1986).
[27] P. Iyer, G. Gompper, and D. A. Fedosov, Non-equilibrium shapes and dynamics of active vesicles, Soft Matter **18**, 6868 (2022).
[28] J. Pécréaux, H.-G. Döbereiner, J. Prost, J.-F. Joanny, and P. Bassereau, Refined contour analysis of giant unilamellar vesicles, The European Physical Journal E **13**, 277 (2004).
[29] A.-Y. Jee, T. Tlusty, and S. Granick, Master curve of boosted diffusion for 10 catalytic enzymes, Proc. Natl. Acad. Sci. USA **117**, 29435 (2020).
[30] L. D. Landau and E. M. Lifshitz, *Statistical Physics* (Elsevier, 1980).
[31] P. Zengel, A. Nguyen-Hoang, C. Schildhammer, R. Zantl, V. Kahl, and E. Horn, μ-slide chemotaxis: a new chamber for long-term chemotaxis studies, BMC cell biology **12**, 1 (2011).
[32] A.-Y. Jee, S. Dutta, Y.-K. Cho, T. Tlusty, and S. Granick, Enzyme leaps fuel antichemotaxis, Proc. Natl. Acad. Sci. USA **115**, 14 (2018).
[33] A.-Y. Jee, Y.-K. Cho, S. Granick, and T. Tlusty, Catalytic enzymes are active matter, Proc. Natl. Acad. Sci. USA **115**, 10812 (2018).
[34] T. M. Squires and S. R. Quake, Microfluidics: Fluid physics at the nanoliter scale, Reviews of modern physics **77**, 977 (2005).
[35] D. Needham and R. S. Nunn, Elastic deformation and failure of lipid bilayer membranes containing cholesterol, Biophysical journal **58**, 997 (1990).
[36] A. Fragasso, N. De Franceschi, P. Stoemmer, E. O. Van Der Sluis, H. Dietz, and C. Dekker, Reconstitution of ultrawide dna origami pores in liposomes for transmembrane transport of macromolecules, ACS nano **15**, 12768 (2021).
[37] P. Schattling, B. Thingholm, and B. Stadler, Enhanced diffusion of glucose-fueled janus particles, Chemistry of Materials **27**, 7412 (2015).
[38] L. M. Jawerth, M. Ijavi, M. Ruer, S. Saha, M. Jahnel, A. A. Hyman, F. Jülicher, and E. Fischer-Friedrich, Salt-dependent rheology and surface tension of protein condensates using optical traps, Physical review letters **121**, 258101 (2018).
